# Prediction of biomass accumulation and tolerance of wheat seedlings to drought and elevated temperatures using hyperspectral imaging

**DOI:** 10.3389/fpls.2024.1344826

**Published:** 2024-02-02

**Authors:** Oksana Sherstneva, Firuz Abdullaev, Dmitry Kior, Lyubov Yudina, Ekaterina Gromova, Vladimir Vodeneev

**Affiliations:** Department of Biophysics, N.I. Lobachevsky State University of Nizhny Novgorod, Nizhny Novgorod, Russia

**Keywords:** hyperspectral imaging, PAM imaging, chlorophyll fluorescence, tolerance, abiotic stress, breeding, *Triticum aestivum* L

## Abstract

Early prediction of important agricultural traits in wheat opens up broad prospects for the development of approaches to accelerate the selection of genotypes for further breeding trials. This study is devoted to the search for predictors of biomass accumulation and tolerance of wheat to abiotic stressors. Hyperspectral (HS) and chlorophyll fluorescence (ChlF) parameters were analyzed as predictors under laboratory conditions. The predictive ability of reflectance and normalized difference indices (NDIs), as well as their relationship with parameters of photosynthetic activity, which is a key process influencing organic matter production and crop yields, were analyzed. HS parameters calculated using the wavelengths in Red (R) band and the spectral range next to the red edge (FR-NIR) were found to be correlated with biomass accumulation. The same ranges showed potential for predicting wheat tolerance to elevated temperatures. The relationship of HS predictors with biomass accumulation and heat tolerance were of opposite sign. A number of ChlF parameters also showed statistically significant correlation with biomass accumulation and heat tolerance. A correlation between HS and ChlF parameters, that demonstrated potential for predicting biomass accumulation and tolerance, has been shown. No predictors of drought tolerance were found among the HS and ChlF parameters analyzed.

## 1 Introduction

Ensuring food security in the context of an ever-growing global population requires an increase in production of agricultural crops, among which wheat occupies a special place, providing up to 20% of the total calories and protein consumed in the world (Shiferaw et al., 2013). To achieve this goal, it is necessary to develop new cultivars that are highly productive and tolerant to external stress (Hossain et al., 2021). To optimize a long and expensive breeding process, classical methods for assessing and selecting promising plant lines are complemented by genotypic and phenotypic studies, which make it possible to identify genotypes that potentially have the necessary economically significant traits.

Integration of advanced genetic technologies such as quantitative trait loci (QTL) mapping, marker-assisted selection and genomic selection into the breeding process has significantly accelerated the development of new cultivars (Alotaibi et al., 2021; Hossain et al., 2021). However, a significant factor limiting the development of breeding is the current gap between the capabilities of genotyping and phenotyping (Grzybowski et al., 2021; Dos Santos et al., 2022; Trono and Pecchioni, 2022). This problem is driving the rapid development of phenotyping methods, among which optical methods occupy a special place, allowing non-invasive acquisition of large amounts of data on various spatial and temporal scales. Among the sensors used in optical phenotyping methods, multi- and hyperspectral, fluorescence and thermal imaging sensors are widely used. Spectral research methods, which allow remotely obtaining highly informative data on the state of plants in both laboratory and field studies at high speed (Perez-Sanz et al., 2017; Sarić et al., 2022), are of particular interest. They are based on the specific nature of the interaction of light of certain ranges in the visible and infrared regions of the spectrum with the plant (Ollinger, 2011). Moreover, changes in tissue structure, content of pigments and other substances, and the activity of physiological processes can cause changes in the spectral properties of the plant (Kim et al., 2021), which provides the potential of multi- and hyperspectral methods in quickly assessing plant responses to changing environmental conditions.

Optical phenotyping is actively used in the early detection of abiotic and biotic stress (Sarić et al., 2022), including drought (Katsoulas et al., 2016) and exposure to elevated temperatures (Venkatesh et al., 2022). Real-time quantitative assessment of plant responses to stressors makes it possible to predict the productivity and stress tolerance of plants long before the harvest stage, which is of great importance for accelerating the selection of promising genotypes in breeding (Gosa et al., 2019). In particular, a fairly large number of works show reliable correlations of canopy reflectance parameters at different stages of wheat development with grain yield under limited water availability (Aparicio et al., 2000; Tattaris et al., 2016; Becker and Schmidhalter, 2017; El-Hendawy et al., 2017; Thapa et al., 2019; Bhandari et al., 2021). The works (S. Pradhan et al., 2012; Bandyopadhyay et al., 2014; Liu et al., 2022) showed high correlation coefficients of yield with wheat reflectance indices at different stages of growth under different irrigation.

Along with drought, elevated temperature is often a negative factor affecting wheat plants. Both problems are intensifying, including due to ongoing climate change (Lippmann et al., 2019; Shahzad et al., 2021; Zahra et al., 2021; Lesk et al., 2022; Sánchez-Bermúdez et al., 2022; Mao et al., 2023; Zahra et al., 2023). A number of works have demonstrated the efficiency of spectral predictors of economically significant traits, which are recorded at the stages of wheat plants development preceding full maturity, under different regimes of water availability and elevated ambient temperatures (Crain et al., 2018; Juliana et al., 2019; Krause et al., 2019).

In addition to the magnitude of changes in spectral indices caused by the action of a stressor, their absolute values determined for plants under optimal growing conditions are also used in predicting crop yields. Thus, a number of works have shown a fairly high ability for a number of vegetation (Tattaris et al., 2016; El-Hendawy et al., 2017; Thapa et al., 2019) and water (El-Hendawy et al., 2017) reflectance indices, as well as their combinations (Hassan et al., 2022) in predicting economically significant traits of wheat grown under optimal water conditions (in irrigated fields).

In addition to the use of reflectance parameters as independent predictors of economically important traits, a number of works have shown the promise of integrating spectral data into multivariate analysis based on genotyping to improve the accuracy of wheat yield prediction (Rutkoski et al., 2016; Sun et al., 2017; Juliana et al., 2019; Lozada and Carter, 2020). Another important direction in the research development is the study of the relationship between phenotypic traits determined using spectral imaging and the activity of physiological processes, primarily photosynthesis, which determines plant productivity. A widely used method for assessing photosynthetic activity is chlorophyll fluorescence (ChlF) recording using Pulse-Amplitude-Modulation (PAM) fluorometry (Maxwell and Johnson, 2000; Baker, 2008; Kalaji et al., 2017; Ni et al., 2019). It is important to note that ChlF parameters determined by the PAM method are effective predictors of economically important traits in wheat under optimal conditions and under the influence of abiotic stress factors (Sharma et al., 2015; Sherstneva et al., 2021; Terletskaya et al., 2021). Our previous work demonstrated the high efficiency of ChlF parameters characterizing transient light-induced processes in the photosynthetic apparatus (PSA) as predictors of biomass accumulation and tolerance to water deficiency and high-temperature stress (Sherstneva et al., 2021).

PAM fluorometry has a number of significant advantages, such as high information content and non-invasiveness, but it has limitations in speed (long time) and throughput. In this regard, a direction of research devoted to the search for the connection between spectral characteristics (which can be quickly determined on a large scale) and chlorophyll fluorescence parameters (which provide information about the activity of photosynthesis) is currently actively developing (Peng et al., 2017; El-Hendawy et al., 2019; Sukhova and Sukhov, 2020). However, knowledge in this area remains incomplete and requires further research.

Another important unsolved problem today is to elucidate the factors that determine the prognostic potential of the studied predictors. To study this issue, assessing the relationship of predictors with both economically important traits and physiological processes seems to be the most effective.

The aim of this work was to analyze the relationship between the spectral characteristics of plants and chlorophyll fluorescence parameters reflecting the activity of photosynthesis, as well as to analyze their predictive potential in relation to such important economic traits as biomass accumulation and tolerance to drought and heat. We conducted laboratory phenotyping under controlled environmental conditions and analyzed the relationship of spectral and chlorophyll fluorescence parameters of 2-week-old wheat seedlings with the rate of biomass accumulation and tolerance to water deficiency and elevated temperatures.

## 2 Materials and methods

### 2.1 Plant material

The experiments were carried out on wheat seedlings (*Triticum aestivum* L.). Eleven cultivars of wheat were used in the study: Lutescens 62, Strubes Schlesischer Grannen, Alen’kaya Uimonskaya, Khludovka, Paradis, Saratovskaya 29, Wachtel, Solo, Kantegirskaya 89, Sibirskaya 12, Naxos (hereinafter, cultivars C1–C11, respectively). Wheat seeds were provided by Federal Research Center N.I. Vavilov All-Russian Institute of Plant Genetic Resources (VIR).

### 2.2 Experiment design

Plants were grown in 1.2 L pots (9 plants per pot, peat soil Peter Peat Agro Black, Peter Peat, Moscow, Russia) under controlled conditions (air temperature 24 °С, relative humidity 50% and a 16 h photoperiod) in a vegetation room. The plants were illuminated using cool white fluorescent lamps L36W/640 (Osram, Munich, Germany); the light intensity was 200 μmol m^−2^ s^−1^.

**Figure 1:**
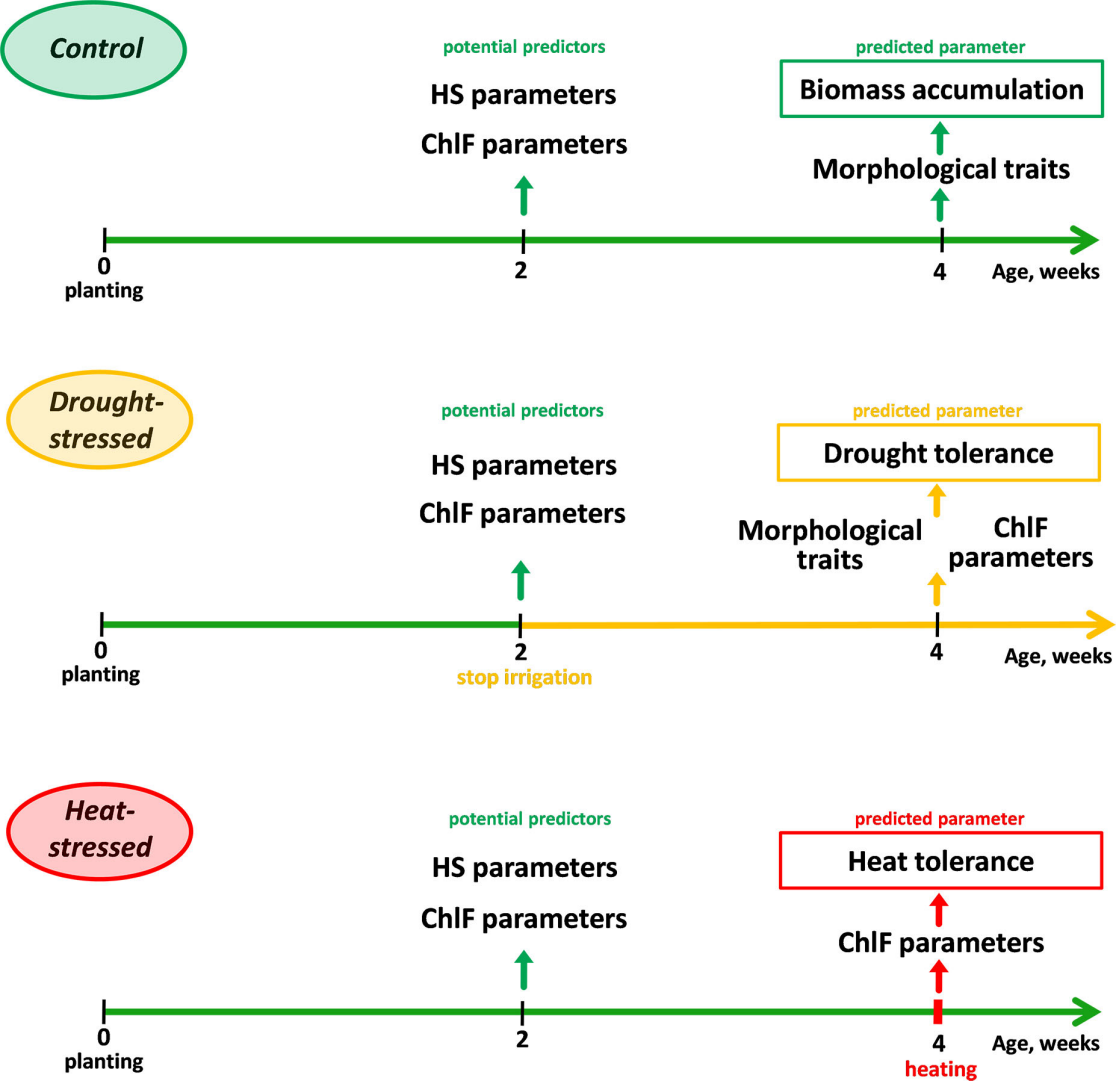
Experiment design for studying wheat biomass accumulation, tolerance to drought, and tolerance to heat stress.

Reflectance and ChlF parameters for all experimental groups were assessed at the age of 2 weeks (**Figure 1**). In the first set of experiments, soil drought stress conditions were applied; for this purpose, irrigation of plants in the experimental group (drought-stressed group) was stopped at the age of 2 weeks. In the control group, irrigation (every 2 days) continued; soil moisture (calculated as $RWC\;=\frac{FW\;-\;DW}{FW}*100\%$ ) was at least 70%. The dynamics of water content in the soil and for the control and drought-stressed groups is shown in **Supplementary Figure 1** in **Supplementary Files**.

Morphometric (length, fresh and dry weight of shoots and roots) and ChlF parameters of plants were measured at the age of 4 weeks to assess tolerance to drought. Drought tolerance index ( $DTI\;=\frac{DW_{drought}}{DW_{control}}*100\%$ ) and residual levels of photosynthetic activity parameters, expressed in % of control ( $Parameter_{resid}\;=\frac{Parameter_{drought}}{Parameter_{control}}*100\%$ ) were used as tolerance indicators. In the second set of experiments, 4-week-old plants grown under conditions of sufficient water availability were subjected to gradual heating of leaves (heat-stressed group) fixed on a hot plate (Microstat-30/80, KB Technom, Yekaterinburg, Russia).

The hot plate temperature increased every 5 minutes from 25 to 55°C in increments of 5°C; the duration of treatment at 55°C was 10 minutes. Infrared images of wheat leaves were acquired using a testo 885 thermal imager (Testo, Lenzkirch, Germany) every 60 s to assess the temperature of wheat leaves and the hot plate. Image analysis was performed using IRSoft software (Testo, Lenzkirch, Germany); regions of interest (ROIs) were placed on wheat leaves and on the hot plate around the leaves.

The tolerance of the photosynthetic apparatus was used as an indicator of wheat tolerance to high-temperature stress. For this purpose, ChlF parameters were recorded using the PAM imaging method simultaneously with heating. The tolerance was expressed quantitatively in two parameters: the residual level of the quantum yield of photochemical reactions of photosystem II (*Φ*_PSII_), calculated as the ratio of the final level of *Φ*_PSII_ at 55°C to the initial level at 25°C, expressed as a percentage ( ${\text{Ф}}_{\text{PSII }resid}\;=\frac{{\text{Ф}}_{\text{PSII}}\left(55{}^{\circ}С\right)}{{\text{Ф}}_{\text{PSII}}\left(25{}^{\circ}С\right)}*100\%$ ), as well as the hot plate temperature at which *Φ*_PSII_ decreased below the initial control value of 25°C (t_dec_).

### 2.3 Morphological traits determination

The length, fresh and dry weight of the roots, shoots and whole 4-week-old plants were used as morphometric parameters of wheat seedlings. Shoots and roots of wheat seedlings were weighed separately using the analytical balance (Explorer EX125D, Ohaus, Parsippany, NJ, USA), then placed in the drying oven for 3 hours at 90°С; after that they were weighed again. Weight was assessed integrally for a pot (9 plants) and calculated for an individual plant. The length of shoots and roots was measured individually for each plant.

### 2.4 PAM imaging

Recording of photosynthetic activity parameters was carried out using a system based on the PAM fluorometry method (Open FluorCam FC 800-O/1010-S, Photon Systems Instruments, Drásov, Czech Republic). Wheat plants were subjected to dark adaptation for 15 minutes, after which the dark (F_0_) and maximum (F_m_) fluorescence yields were determined. Actinic light (AL, cool white light, 200 µmol m^−2^ s^−1^) was then turned on, and the current (F) and maximum (F_m_’) fluorescence yields were determined every 30 s for 15 minutes.

The parameters F_m_ and F_m_′ were measured using a saturation pulse (cold white light, 4000 µmol m^−2^ s^−1^, 800 ms duration, 6500 K). Based on the recorded values, parameters of photosynthetic activity such as the quantum yield of photochemical reactions of photosystem II (*Φ*_PSII_) and non-photochemical quenching of chlorophyll fluorescence (NPQ) were calculated by a program integrated into the recording system, and their dynamics, induced by a change in the illumination mode, were analyzed. *Φ*_PSII_ and NPQ values were calculated using the equations: *Φ*_PSII_ = (F_m_′ − F)/F_m_′ and NPQ = (F_m_ − F_m_′)/F_m_′ (Maxwell and Johnson, 2000), where F_m_ is the maximum fluorescence yield of chlorophyll after dark adaptation, F_m_′ is the maximum fluorescence yield under lighting, F is the current fluorescence level under lighting.

**Figure 2:**
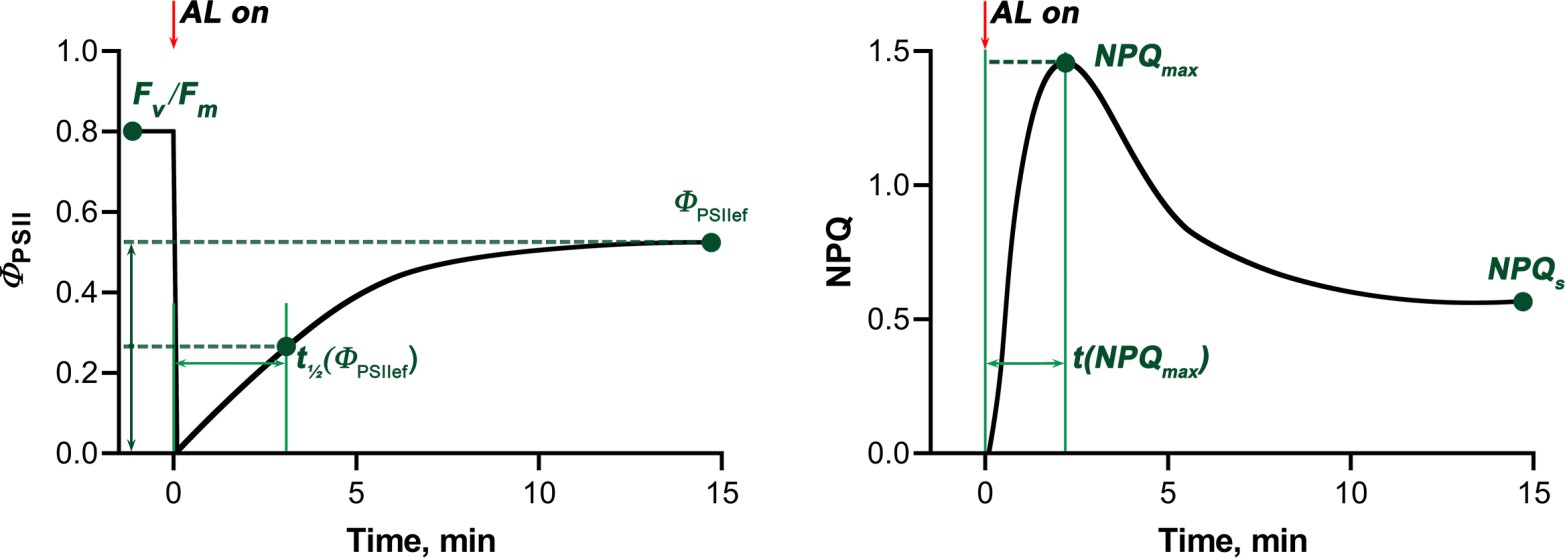
Scheme illustrating standard light-induced *Φ*_PSII_ and NPQ curves and characteristic values used to quantify photosynthetic activity. The red arrows mark the moment when the actinic light was switched on (AL on). F_v_/F_m_ - maximum quantum yield of photochemical reactions of photosystem II, *Φ*_PSIIef_ - steady-state level of photosystem II quantum yield under AL, t_1/2_(*Φ*_PSIIef_) - time for the *Φ*_PSII_ value to reach ½ *Φ*_PSIIef_ after switching on AL, NPQ_max_ - maximum value of non-photochemical fluorescence quenching after switching on AL, t(NPQ_max_) - time for the NPQ value to reach NPQ_max_ after switching on AL, NPQ_s_ - steady-state NPQ level under AL.

The following quantitative characteristics of the light-induced dynamics of *Φ*_PSII_ and NPQ were analyzed (**Figure 2**): maximum quantum yield of photochemical reactions of photosystem II, determined after dark adaptation (F_v_/F_m_), steady-state level of photosystem II quantum yield under AL (*Φ*_PSIIef_), time for the *Φ*_PSII_ value to reach ½ *Φ*_PSIIef_ after switching on AL (t_1/2_(*Φ*_PSIIef_)), maximum value of non-photochemical fluorescence quenching after switching on AL (NPQ_max_), time for the NPQ value to reach NPQ_max_ after switching on AL (t(NPQ_max_)), and steady-state NPQ level under AL (NPQ_s_) (Sherstneva et al., 2021).

In experiments with gradual heating of leaves, plants were also adapted to darkness within 15 minutes. Then the initial parameters (F_0_ and F_m_) were determined, AL was turned on, and ChlF parameters were recorded every 60 s for 40 minutes.

### 2.5 Hyperspectral imaging

Hyperspectral images of plants were obtained using the Specim IQ hyperspectral camera (Specim, Spectral Imaging Ltd., Oulu, Finland). Images of wheat leaves were separated from the background, then ROIs were placed (each ROI included the whole aboveground part of all plants in the pot above 3 cm from the soil); one reflectance spectrum was integrally obtained from each ROI. The resulting reflectance spectra of shoots in the range of 400-800 nm in steps of 3 nm were normalized by the reflectance spectrum of the white standard. Normalized difference indices (NDIs) were also calculated for each combination of wavelengths according to the equation:


$$NDI=\;\frac{I_{\lambda_{1}}-\;I_{\lambda_{2}}}{I_{\lambda_{1}}+\;I_{\lambda_{2}}},$$

where $I_{\lambda_{1}}$ and $I_{\lambda_{2}}$ are the intensities of the reflectance at the wavelengths $\lambda_{1}$ and $\lambda_{2}$ , respectively.

NDIs were presented as heat maps.

### 2.6 Statistics

Statistical processing of the results was carried out using GraphPad Prism (GraphPad Software Inc., San Diego, CA, USA) and Microsoft Excel (Microsoft Corporation, Redmond, WA, USA). The results are presented as average curves with standard errors of the mean (SEM), average values with SEM, heat maps of parameters, as well as spectra and heat maps of Pearson correlation coefficients (r). The probability value (p-value) was used to assess the statistical significance of the result; p< 0.05 was considered significant. The normality of data distribution was assessed using the Kolmogorov–Smirnov test. The one-way analysis of variance (ANOVA) followed by Tukey’s test was used.

At the age of 2 weeks, 4 pots of 9 plants for each cultivar were assessed in each experimental group (control, drought-stressed, heat-stressed). Weight was assessed integrally for a pot (9 plants) and calculated for an individual plant (*n* = 4). Shoot and root length were measured for individual plants. The chlorophyll fluorescence parameters were registered for 5 plants per pot (*n* = 20 for 2-week-old plants; *n* = 10 for 4-week-old plants in each experimental group).

## 3 Results

### 3.1 Wheat parameters under control and stress conditions

#### 3.1.1 Morphological, hyperspectral, and ChlF parameters under control conditions

**Figure 3:**
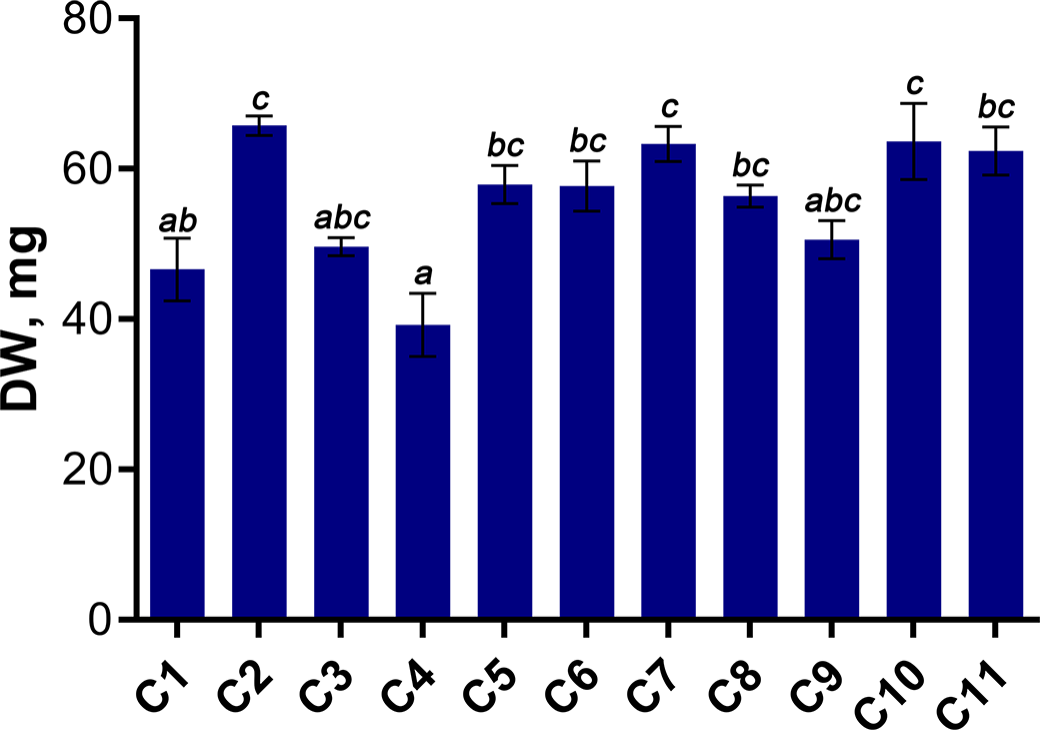
Dry weight (DW) of 4-week-old wheat seedlings. Data are presented as means with SE bars (*n = 4*). Significant differences between the cultivars are indicated by different letters (ANOVA followed by Tukey’s test, p< 0.05). Values with the same letters are not significantly different.

In this study, the morphometric parameters of 4-week-old wheat seedlings, including length, fresh and dry weight of shoots and roots were assessed (**Figure 3**, **Table S1**). Whole plant dry weight (DW) ranged from 29.4 to 77.05 mg; subsequently, this parameter was used as the most informative parameter reflecting the accumulation of biomass by wheat seedlings.

Chlorophyll fluorescence (ChlF) and hyperspectral characteristics (HS parameters), which were further analyzed as potential predictors of biomass accumulation and tolerance to drought and heat stress, were determined in 2-week-old wheat seedlings. ChlF parameters were recorded using the PAM imaging method (**Table 1**). To assess the functioning of the PSA, such parameters of chlorophyll fluorescence as stationary levels of the quantum yield of photochemical reactions of photosystem II and non-photochemical quenching of fluorescence in light and in the dark, as well as parameters characterizing the rate of transition processes caused by the dark-light transition were analyzed (**Figure 2**).

**Table 1:** ChlF parameters of 2-week-old wheat plants.

Cultivar	F_v_/F_m_	*Φ*_PSIIef_	NPQ_s_	t_1/2_(*Φ*_PSIIef_)	NPQ_max_	t(NPQ_max_)
C1	0.812 ± 0.001	0.456 ± 0.010	0.760 ± 0.027	229.2 ± 10.6	1.727 ± 0.031	219.0 ± 7.6
C2	0.813 ± 0.001	0.558 ± 0.008	0.532 ± 0.019	167.2 ± 8.1	1.415 ± 0.043	160.5 ± 7.3
C3	0.810 ± 0.001	0.506 ± 0.011	0.704 ± 0.025	198.4 ± 12.3	1.718 ± 0.036	195.0 ± 10.1
C4	0.810 ± 0.001	0.499 ± 0.010	0.742 ± 0.036	231.5 ± 8.9	1.739 ± 0.028	226.5 ± 7.7
C5	0.808 ± 0.001	0.607 ± 0.006	0.442 ± 0.017	173.3 ± 8.8	1.407 ± 0.050	175.3 ± 6.2
C6	0.808 ± 0.001	0.513 ± 0.013	0.775 ± 0.045	154.5 ± 11.2	1.701 ± 0.051	186.0 ± 7.1
C7	0.820 ± 0.001	0.568 ± 0.007	0.589 ± 0.020	197.2 ± 9.8	1.846 ± 0.040	190.5 ± 5.9
C8	0.812 ± 0.001	0.506 ± 0.013	0.659 ± 0.025	113.7 ± 5.6	1.306 ± 0.036	136.5 ± 10.1
C9	0.805 ± 0.001	0.515 ± 0.009	0.670 ± 0.033	186.4 ± 10.5	1.608 ± 0.030	220.0 ± 9.1
C10	0.812 ± 0.001	0.577 ± 0.007	0.471 ± 0.018	104.9 ± 6.4	1.244 ± 0.033	147.0 ± 6.5
C11	0.810 ± 0.001	0.571 ± 0.009	0.573 ± 0.026	128.5 ± 6.6	1.490 ± 0.031	144.0 ± 6.0

F_v_/F_m_ - maximum quantum yield of photochemical reactions of photosystem II, Φ_PSIIef_ - steady-state level of photosystem II quantum yield under AL, t_1/2_(Φ_PSIIef_) - time for the Φ_PSII_ value to reach ½ Φ_PSIIef_ after switching on AL, NPQ_max_ - maximum value of non-photochemical fluorescence quenching after switching on AL, t(NPQ_max_) - time for the NPQ value to reach NPQ_max_ after switching on AL, NPQ_s_ - steady-state NPQ level under AL. Data are represented as means ± SEM (n = 20). Significant differences between the cultivars are indicated by different letters (ANOVA followed by Tukey’s test, p< 0.05). Values with the same letters are not significantly different.

**Figure 4:**
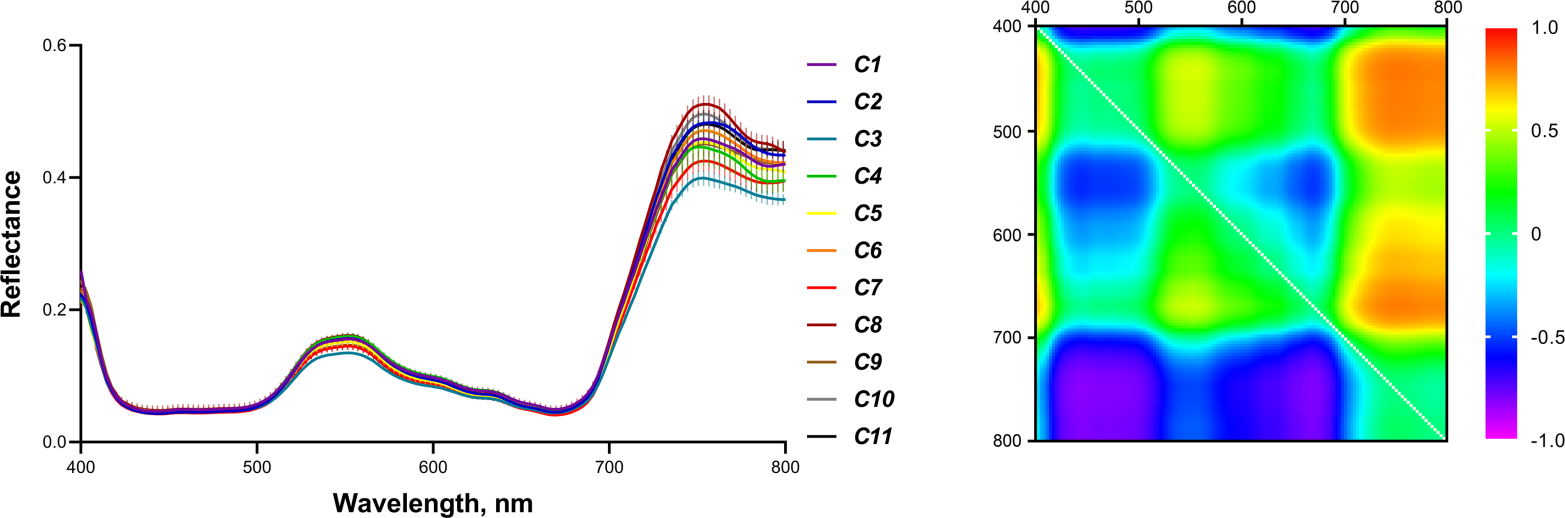
Reflectance spectra of 2-week-old wheat seedlings of 11 cultivars (means with SE bars) and an example of an NDIs heat map (cultivar C1).

Along with determining ChlF parameters, spectral characteristics of 2-week-old seedlings were recorded. To search for spectral parameters that can act as predictors of biomass accumulation and wheat tolerance to soil moisture deficiency and heating, the reflectance spectra of the studied plants were obtained using hyperspectral imaging. **Figure 4** shows the average reflectance spectra of shoots of 2-week-old wheat seedlings of 11 cultivars. The reflectance spectrum has typical minimums in the blue and red regions, a sharp rise in the red edge band, and high values in the near-infrared (NIR) region. **Figure 4** shows an example of a heat map of normalized difference indices (NDIs). NDIs heat maps for all studied cultivars are presented in **Supplementary Figure 2** in **Supplementary Files**. Subsequently, the absolute values of the intensity of the reflected light (normalized to the intensity of the light reflected by the white standard) in the range of 400-800 nm in steps of 3 nm and the entire set of NDIs calculated for this wavelength range were used to search for correlations of the reflectance parameters of young plants with dry weight and stress tolerance of plants of later age.

#### 3.1.2 Relationship between HS and ChlF parameters

**Figure 5:**
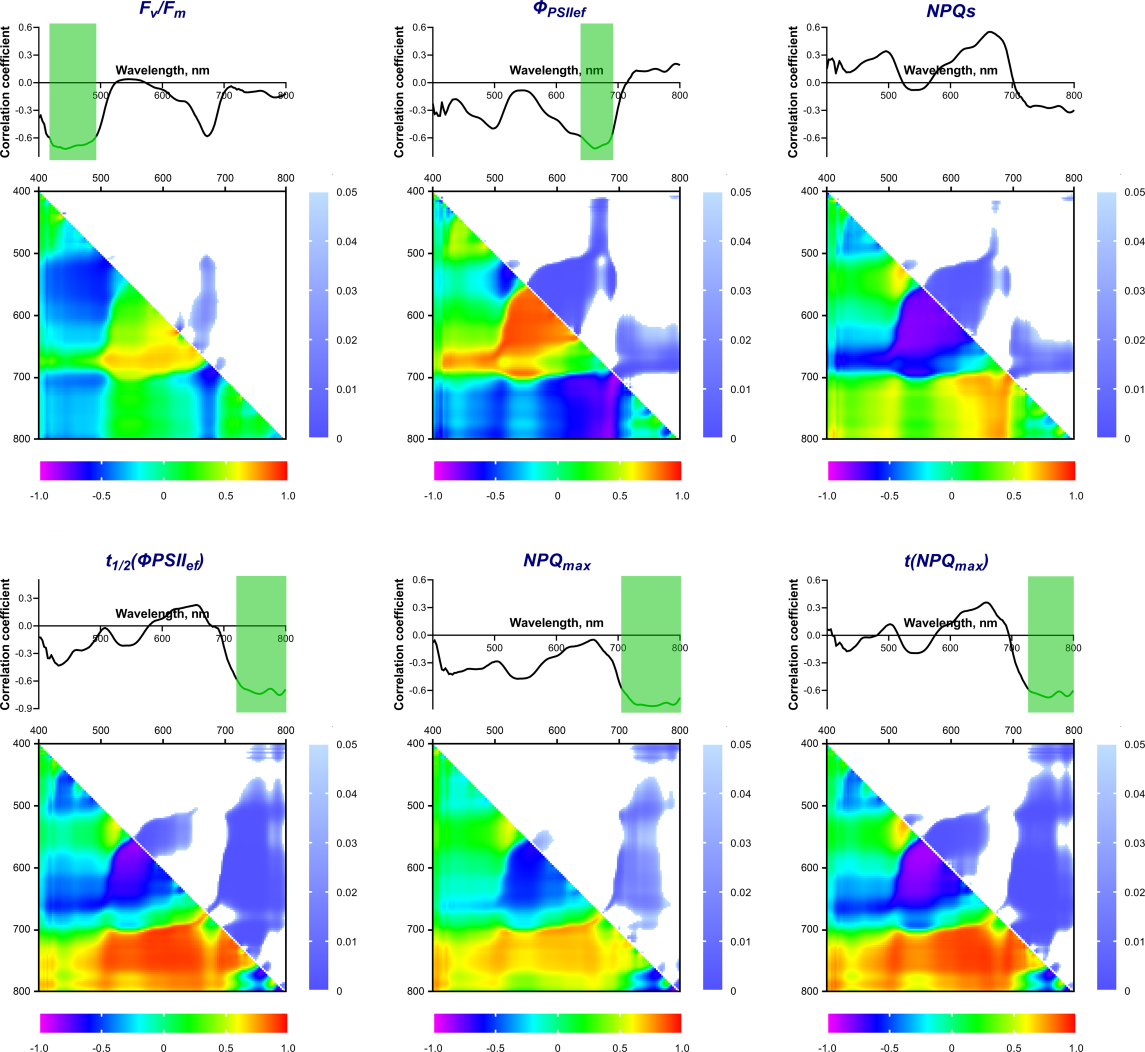
Spectra and heat maps of Pearson correlation coefficients between HS and ChlF parameters of 2-week-old wheat plants. Areas of the spectra with statistically significant correlation are highlighted in green. Heat maps show correlation coefficients (bottom left) and p-values (top right).

The next step was to study the possible connection between reflectance properties and the activity of photosynthetic processes. For this purpose, correlation coefficients were calculated between the intensity of reflected light at a certain wavelength in the range of 400-800 nm and the ChlF parameters (**Figure 5**). F_v_/F_m_, *Φ*_PSIIef_ and NPQ_s_ had maximum values of Pearson correlation coefficients (r) with reflectance intensity in the blue (B) and red (R) regions of the spectrum. These coefficients were negative for F_v_/F_m_ and *Φ*_PSIIef_ and positive for NPQ_s_. A statistically significant (p < 0.05) relationship was detected for F_v_/F_m_ in the B range (420-490 nm, the correlation coefficient reached –0.72); there was no significant correlation in the R range, but a tendency towards a negative relationship was observed. On the contrary, *Φ*_PSIIef_ was significantly negatively correlated with reflectance intensity in the R range (640-690 nm); r reached –0.72. No statistically significant correlation was found for NPQ_s_.

The maximum values of the correlation coefficients for the parameters t_1/2_(*Φ*_PSIIef_), NPQ_max_, and t(NPQ_max_) occurred in the far-red-NIR (FR-NIR) range (710-800 nm). The wavelength ranges with significant correlation were: 720-800 nm for t_1/2_(*Φ*_PSIIef_) (r reached –0.75), 710-800 nm for NPQ_max_ (r reached –0.77), and 730-800 nm for t(NPQ_max_) (r reached –0.68).

A correlation analysis of the relationship between ChlF parameters and NDIs, calculated using all combinations of recorded wavelengths in the range of 400-800 nm, was also carried out. **Figure 5** shows heat maps of correlation coefficients and statistical significance level values. It was shown that F_v_/F_m_ correlates significantly (p < 0.05) (max r = –0.68) with NDIs calculated using λ_1_ 650-685 (hereinafter referred to as R range); λ_2_ 510-620 nm. *Φ*_PSIIef_ had a wider significant region in this range (NDI(λ_1_ 560-650; λ_2_ 500-610) and NDI(λ_1_ 650-690; λ_2_ 410-560), max r = –0.88 and –0.83, respectively). In addition, a small significant region was noted in the FR-NIR range: NDI(λ_1_ 700-800; λ_2_ 620-700), max r = 0.84. NPQ_s_ also demonstrated a similar relationship. Transient ChlF parameters showed a significant relationship with the wider FR-NIR range: NDI(λ_1_ 700-800; λ_2_ 450-710 for t_1/2_(*Φ*_PSIIef_) (max r = –0.92), NDI(λ_1_ 700-780; λ_2_ 450-700) for NPQ_max_ (max r = –0.72), NDI(λ_1_ 700-800; λ_2_ 400-720 for t(NPQ_max_) (max r = –0.92). In addition, t_1/2_(*Φ*_PSIIef_) and t(NPQ_max_) had a high significant correlation with NDIs in the R range: NDI(λ_1_ 560-650; λ_2_ 510-570) for t_1/2_(*Φ*_PSIIef_), NDI(λ_1_ 560-650; λ_2_ 510-610) for t(NPQ_max_) (max r = 0.83 and 0.86, respectively).

#### 3.1.3 Drought stress

Along with recording parameters under control conditions, the tolerance of wheat plants to abiotic stressors was assessed. The residual level of dry weight of the whole plants was assessed as a criterion for the tolerance of wheat seedlings to drought (**Table 2**, **Supplementary Table 2**). An additional parameter reflecting the degree of suppression of physiological processes in wheat under water deficiency was the residual level of activity and integrity of the PSA, which was determined by the residual levels of such ChlF parameters as F_v_/F_m_ and *Φ*_PSIIef_ (**Table 2**).

**Table 2:** Residual values of dry weight and ChlF parameters, expressed in % of control, after 14-day drought stress of wheat plants.

Cultivar	Residual DW plant, %	Residual F_v_/F_m_, %	Residual *Φ*_PSIIef_, %
C1	81.9 ± 0.4^ab^	93.7 ± 2.4^abc^	60.5 ± 7.6^abc^
C2	68.0 ± 3.3^a^	89.6 ± 4.5^ab^	28.6 ± 5.6^ab^
C3	74.8 ± 4.4^ab^	97.7 ± 1.3^abc^	46.5 ± 6.7^abc^
C4	107.9 ± 6.3^c^	99.3 ± 1.9^bc^	69.2 ± 5.4^c^
C5	94.9 ± 6.9^bc^	97.7 ± 1.1^abc^	48.5 ± 3.8^abc^
C6	90.0 ± 2.1^abc^	89.4 ± 3.6^a^	49.0 ± 11.7^abc^
C7	74.0 ± 3.7^ab^	98.8 ± 0.9^bc^	53.1 ± 3.3^abc^
C8	80.7 ± 5.2^ab^	99.4 ± 0.9^bc^	41.4 ± 6.2^abc^
C9	82.6 ± 6.2^ab^	102.6 ± 0.8^c^	66.1 ± 1.9^bc^
C10	67.9 ± 5.0^a^	88.9 ± 1.8^a^	26.8 ± 2.9^a^
C11	92.2 ± 3.1^abc^	102.2 ± 1.4^c^	69.3 ± 7.8^c^

DW plant - dry weight of a whole plant, F_v_/F_m_ - maximum quantum yield of photochemical reactions of photosystem II, Φ_PSIIef_ - steady-state level of photosystem II quantum yield under AL. Data are presented as means ± SEM (n = 10). Significant differences between the cultivars are indicated by different letters (ANOVA followed by Tukey’s test, p< 0.05). Values with the same letters are not significantly different.

Residual DW of drought-stressed plants (14-day drought), which was subsequently used as the main indicator of tolerance (drought tolerance index), varied significantly between cultivars. At the same time, the relative water content (RWC) of control plants varied from 85.8 to 89.5% and averaged 87.9 ± 0.2%; RWC in drought-stressed plants varied from 45.6 to 81.2 and averaged 70.2 ± 1.5%. The dynamics of RWC in control and drought-stressed plants is shown in **Supplementary Figure 3** in **Supplementary Files**. RWC values did not differ significantly between the studied cultivars, both in control and under conditions of soil moisture deficiency. Residual levels of ChlF parameters, reflecting the sensitivity of photosynthetic processes to water deficiency, varied significantly (**Table 2**). At the same time, the residual level of *Φ*_PSIIef_ significantly correlated with the drought tolerance index (**Table 3**), which indicates the potential possibility of using this parameter as an indicator of stress and plant sensitivity to water deficiency.

**Table 3:** Correlation coefficients between the drought tolerance index and residual levels of ChlF parameters in wheat plants affected by a 14-day drought.

	Drought tolerance index	Residual F_v_/F_m_, %	Residual *Φ*_PSIIef_, %
Drought tolerance index		0.43	**0.72**
Residual F_v_/F_m_, %	0.18		**0.72**
Residual *Φ*_PSIIef_, %	**0.01**	**0.01**	

Statistically signiﬁcant correlation coefﬁcients and the corresponding P-values (two-tailed, p< 0.05) are in bold.

#### 3.1.4 Heat stress

**Figure 6:**
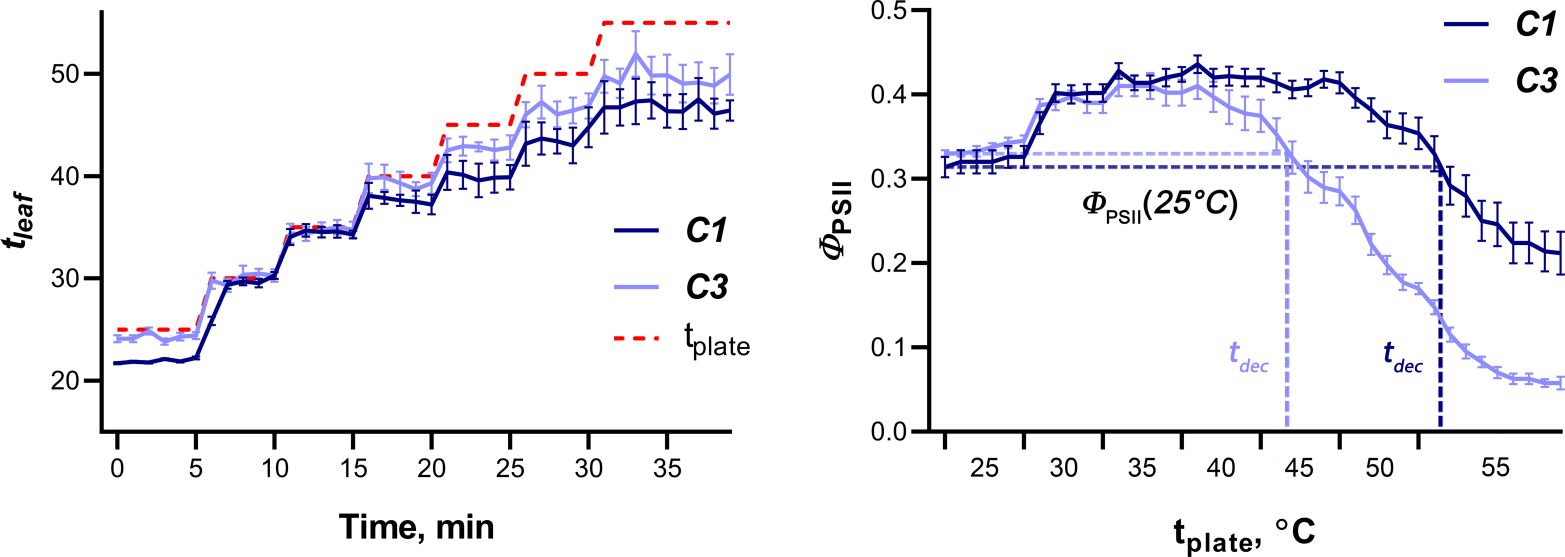
Dynamics of wheat leaf temperature (left) and *Φ*_PSII_ (right) in plants of tolerant (C1) and sensitive to heat (C3) cultivars. Data are presented as means with SE bars. Red dashed line on the left graph indicates the temperature of a hot plate.

To study the tolerance of wheat seedlings to elevated temperatures, gradual heating of wheat leaves was carried out using a hot plate; the dynamics of *Φ*_PSII_ was recorded simultaneously (**Figure 6**). The temperature of the hot plate and leaves was recorded using a thermal imager. The dynamics of photosynthesis activity induced by gradual heating was similar for different cultivars, but the quantitative parameters varied significantly. **Figure 6** shows an example of the average dynamics of leaf temperature and *Φ*_PSII_ in plants of cultivars contrasting in sensitivity to elevated temperature (C1 and C3, which were later classified as tolerant and sensitive to heat, respectively). It was shown that *Φ*_PSII_ increased as the hot plate temperature increased from 25°C to values ranging from 35.5 (C2) to 41°C (C1 and C9); with a further increase in temperature, *Φ*_PSII_ gradually decreased and reached values in the range from 0.09 (C3) to 0.23 (C8) at a final hot plate temperature of 55°C. Two parameters were used as criteria for the tolerance of wheat seedlings to heat: the temperature of the hot plate, at which *Φ*_PSII_ decreased below the initial level at 25°C (t_dec_), as well as the residual level of *Φ*_PSII_ at 55°C, expressed as a percentage of the initial level at 25° (*Φ*_PSII_ resid). The average values of t_dec_ and *Φ*_PSII_ resid for all studied cultivars are shown in **Table 4**. Correlation analysis showed a strong positive relationship between these parameters of heat tolerance (r = 0.84, p = 0.001), therefore, only t_dec_ was chosen for further analysis.

**Table 4:** Parameters of heat tolerance of 4-week-old wheat seedlings of 11 cultivars.

Cultivar	t_dec_	*Φ*_PSII_ *_resid_*, %
C1	53.5 ± 1.0^cd^	66.9 ± 7.6^d^
C2	49.0 ± 0.6^ab^	44.7 ± 1.8^abcd^
C3	46.0 ± 1.3^ab^	23.1 ± 6.6^a^
C4	54.0 ± 0.6^d^	64.8 ± 7.4^cd^
C5	49.5 ± 0.5^bc^	29.9 ± 3.3^ab^
C6	47.5 ± 1.1^ab^	41.6 ± 6.1^abcd^
C7	47.5 ± 1.4^ab^	50.1 ± 8.2^abcd^
C8	48.5 ± 1.0^ab^	58.0 ± 6.5^bcd^
C9	55.0 ± 0.0^d^	68.3 ± 6.8^d^
C10	45.5 ± 0.5^ab^	25.6 ± 2.3^a^
C11	45.0 ± 0.8^a^	35.3 ± 7.2^abc^

Data are presented as means ± SEM. Significant differences between the cultivars are indicated by different letters (ANOVA followed by Tukey’s test, p< 0.05). Values with the same letters are not significantly different.

### 3.2 Predictors of biomass accumulation

#### 3.2.1 HS parameters

A correlation analysis of the relationship between the dry weight of 4-week-old wheat seedlings and the HS parameters of the shoots of 2-week-old plants was carried out to search for predictors of biomass accumulation in plants under optimal temperature and water conditions.

**Figure 7:**
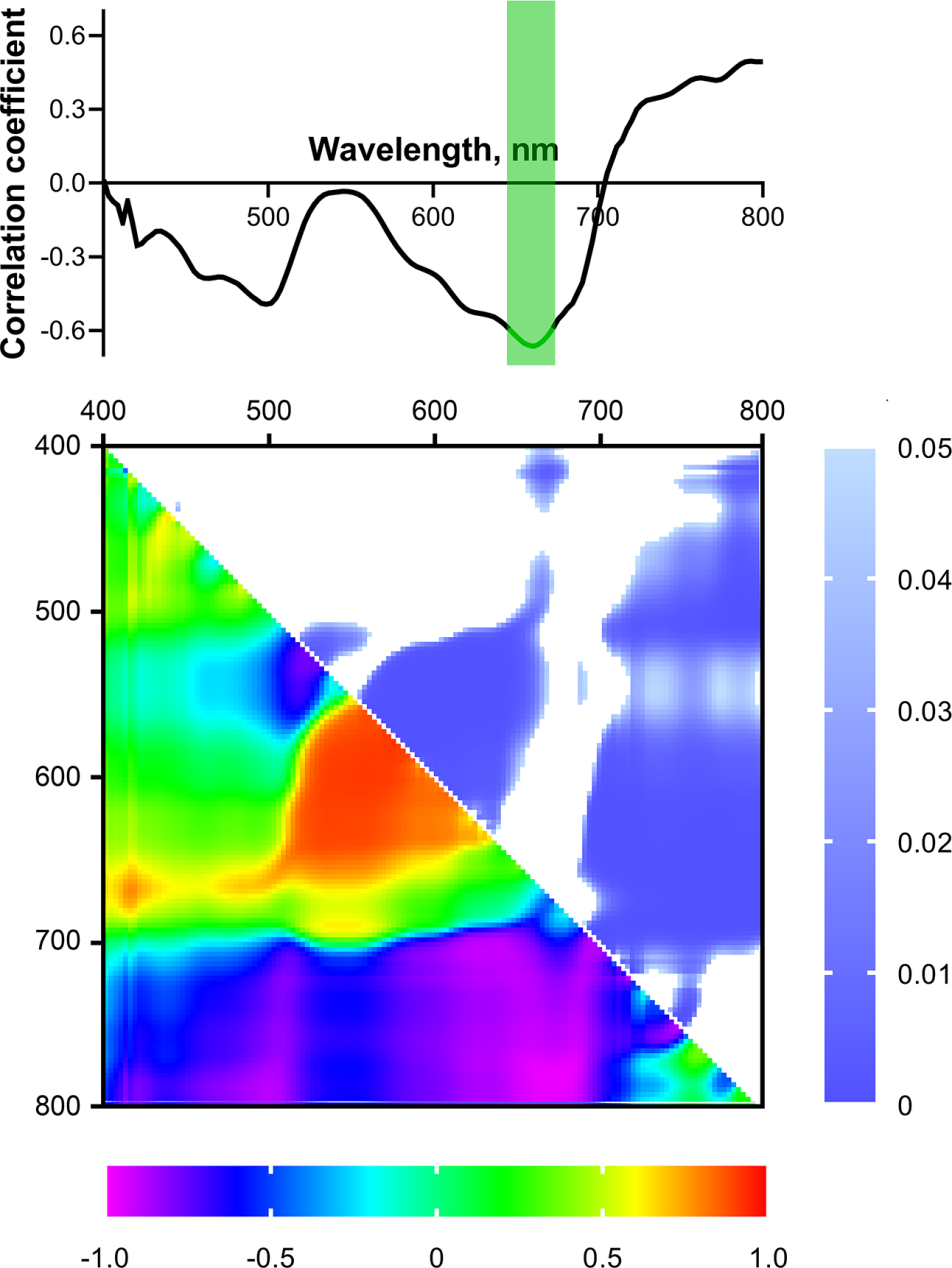
Spectrum and heat map of Pearson correlation coefficients between HS parameters of 2-week-old and DW of 4-week-old wheat plants. Area of the spectrum with statistically significant correlation is highlighted in green. Heat map shows correlation coefficients (bottom left) and p-values (top right).

**Figure 7** shows the spectrum of correlation coefficients of dry weight of 4-week-old seedlings with the reflectance intensity (normalized to the reflectance intensity of the white standard) of 2-week-old seedlings in the wavelength range from 400 to 800 nm. The highest correlation coefficients for these parameters were in the R (650-670 nm) and B (480-510 nm) bands (negative correlations), as well as in the FR-NIR range (750-800 nm, positive correlation). A statistically significant (p< 0.05) correlation was observed only in the R range of spectrum; the Pearson correlation coefficient reached –0.67. In B and FR-NIR bands, r reached –0.49 and 0.5, respectively; however, the correlation was not significant.

Next, NDIs calculated for all combinations of wavelengths in the range from 400 to 800 nm were considered as potential predictors of biomass accumulation. **Figure 7** shows a heat map of correlation coefficients between NDIs of shoots of 2-week-old wheat seedlings and DW of 4-week-old ones. The highest correlation coefficients were observed for NDIs calculated using wavelengths in the R and FR-NIR bands. Pearson correlation coefficients reached 0.91 and –0.97 (p< 0.05) for NDI(λ_1_ 580-670; λ_2_ 520-600) and NDI(λ_1_ 710-800; λ_2_ 400-700), respectively.

#### 3.2.2 ChlF parameters

An analysis of the relationship between ChlF parameters of 2-week-old wheat seedlings and the dry weight of 4-week-old plants showed that a number of parameters of photosynthetic activity at an early age correlate with the biomass accumulation at a later age (**Table 5**). In particular, a significant correlation of DW with stationary parameters of photosynthetic activity in a state adapted to light was shown (a positive correlation with *Φ*_PSII_ (r = 0.74) and a negative correlation with NPQ (r = –0.70)). In addition, a number of parameters reflecting transient processes caused by changes in lighting conditions were negatively correlated with the biomass accumulation [r = –0.69 for t_1/2_(*Φ*_PSIIef_) and r = –0.77 for t(NPQ_max_)].

**Table 5:** Correlation coefficients between ChlF parameters of 2-week-old wheat seedlings and the dry weight of 4-week-old plants.

	F_v_/F_m_	*Φ*_PSIIef_	t_1/2_(*Φ*_PSIIef_)	NPQ_max_	t(NPQ_max_)	NPQ_s_
*r* p	*0.36* 0.27	***0.74*** **0.01**	***-0.69*** **0.02**	*-0.49* 0.13	***-0.77*** **0.005**	***-0.70*** **0.02**

Statistically signiﬁcant correlation coefﬁcients and the corresponding p-values (two-tailed, p< 0.05) are in bold.

### 3.3 Predictors of drought tolerance

#### 3.3.1 HS parameters

**Figure 8:**
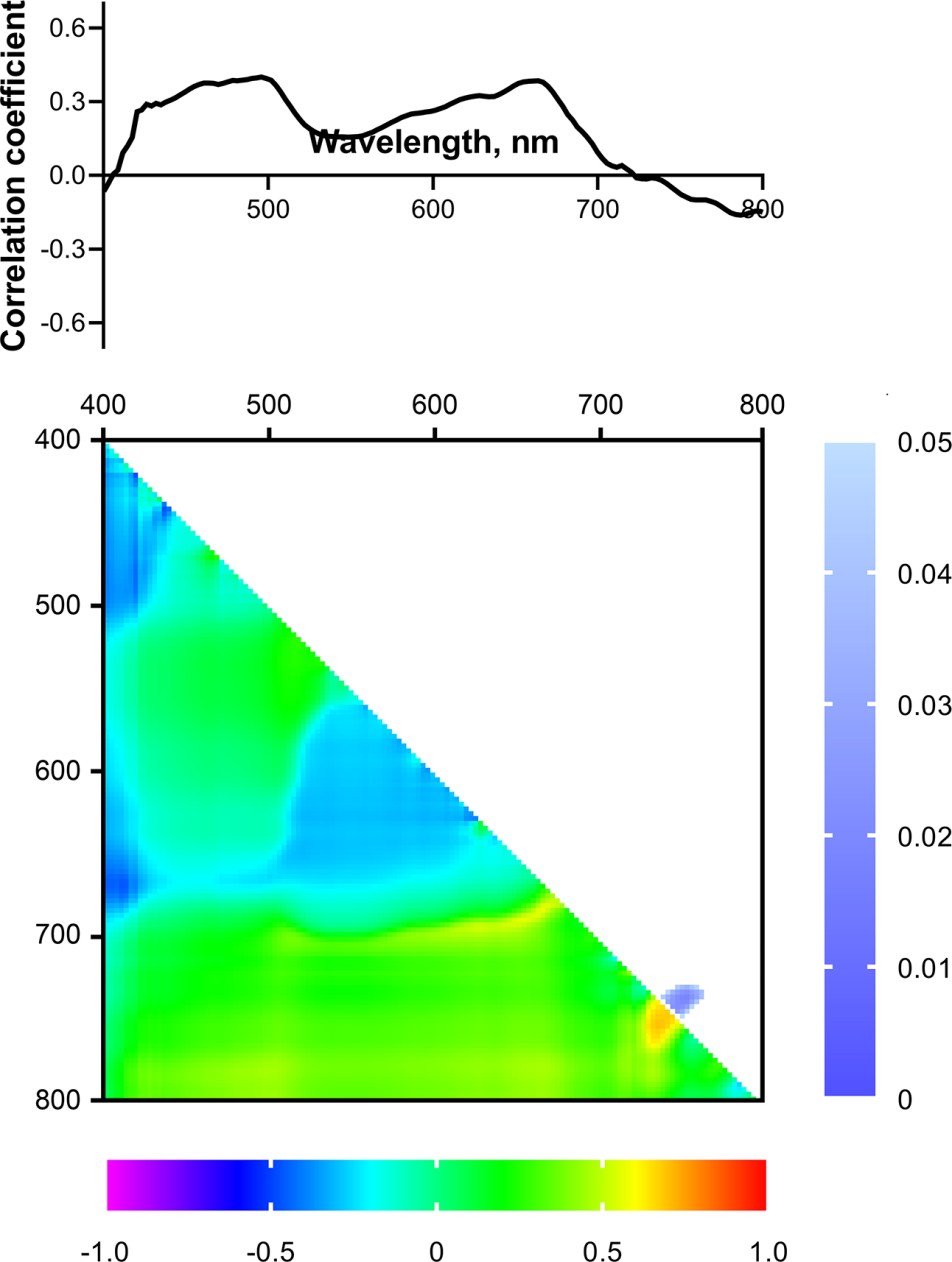
Spectrum and heat map of Pearson correlation coefficients between HS parameters of 2-week-old wheat plants and the drought tolerance index of 4-week-old wheat plants. Heat map shows correlation coefficients (bottom left) and p-values (top right).

**Figure 9:**
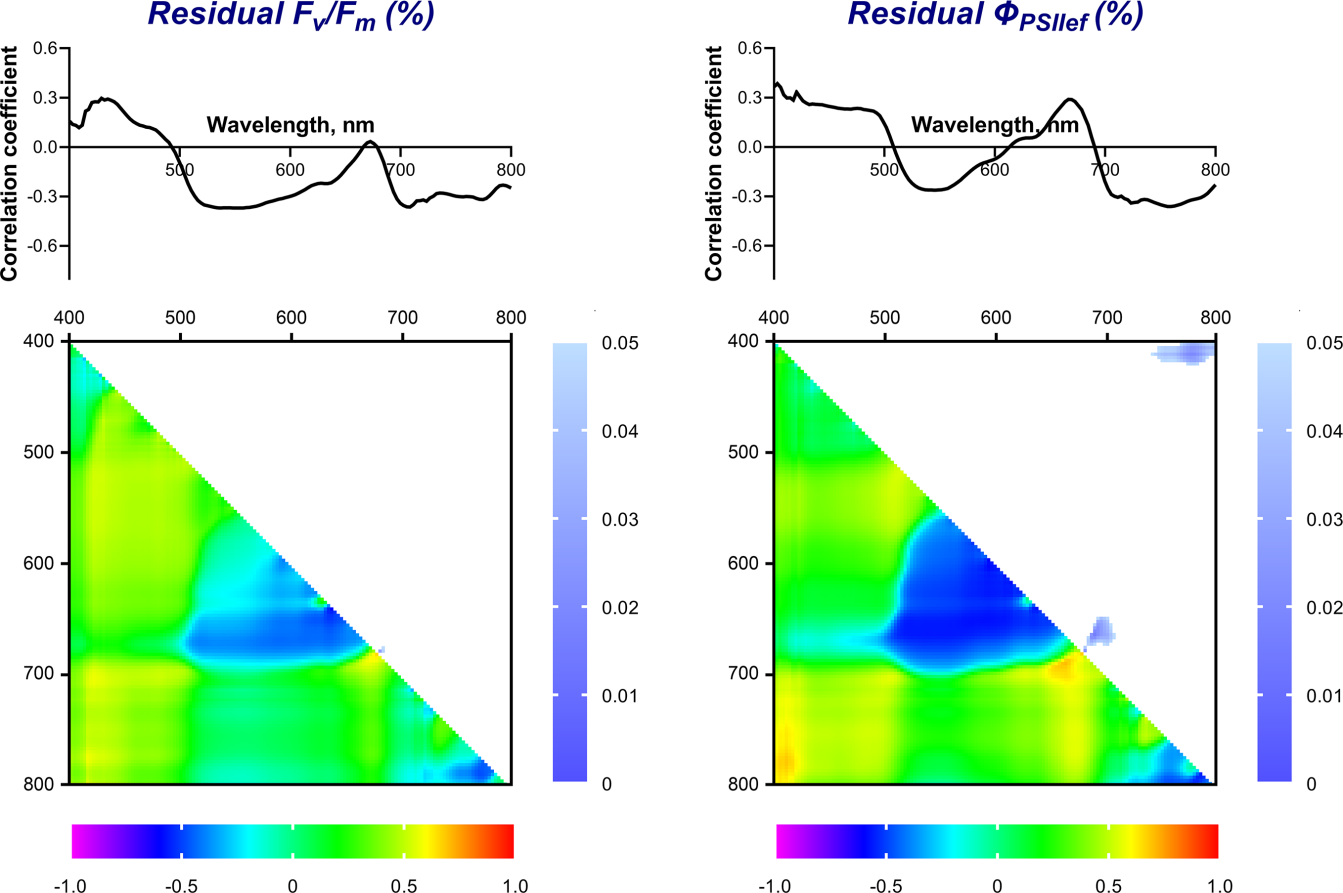
Spectra and heat maps of Pearson correlation coefficients between HS of 2-week-old and residual levels of ChlF parameters of 4-week-old wheat plants. Heat maps show correlation coefficients (bottom left) and p-values (top right).

To search for predictors of drought tolerance of wheat, a correlation analysis between the reflectance parameters of 2-week-old plants and the drought tolerance index, estimated by the residual DW of 4-week-old drought-stressed plants (in % of control) was carried out. However, no significant relationship was shown between the drought tolerance index and the reflectance of 2-week-old seedlings in the wavelength range from 400 to 800 nm (**Figure 8**). A similar result was obtained for the residual levels of ChlF parameters, reflecting the tolerance of the PSA to water deficiency (**Figure 9**).

Statistically significant correlation of the NDIs (considered as potential predictors of drought tolerance) with the drought tolerance index and residual levels of ChlF parameters were also not observed (**Figures 8**, **9**). We did not take significant areas of heat maps for the drought tolerance index and residual *Φ*_PSII_ into further analysis due to their small size and low correlation coefficients.

#### 3.3.2 ChlF parameters

As in the case of spectral characteristics, correlation analysis of the relationship between ChlF parameters and drought tolerance also did not show a significant correlation (**Table 6**).

**Table 6:** Correlation coefficients between ChlF parameters of 2-week-old wheat seedlings and drought tolerance of 4-week-old plants.

		F_v_/F_m_	*Φ*_PSIIef_	t_1/2_(*Φ*_PSIIef_)	NPQ_max_	t(NPQ_max_)	NPQ_s_
*Residual DW*	*r* p	*-0.45* 0.16	*-0.13* 0.69	*0.32* 0.34	*0.26* 0.44	*0.36* 0.27	*0.31* 0.35
*Residual F_v_/F_m_*	*r* p	*-0.16* 0.65	*-0.39* 0.91	*0.20* 0.56	*0.22* 0.51	*0.20* 0.57	*0.1* 0.77
*Residual Φ_PSII_*	*r* p	*-0.29* 0.39	*-0.34* 0.31	*0.52* 0.10	*0.59* 0.06	*0.58* 0.06	*0.49* 0.13

### 3.4 Predictors of heat tolerance

#### 3.4.1 HS parameters

**Figure 10:**
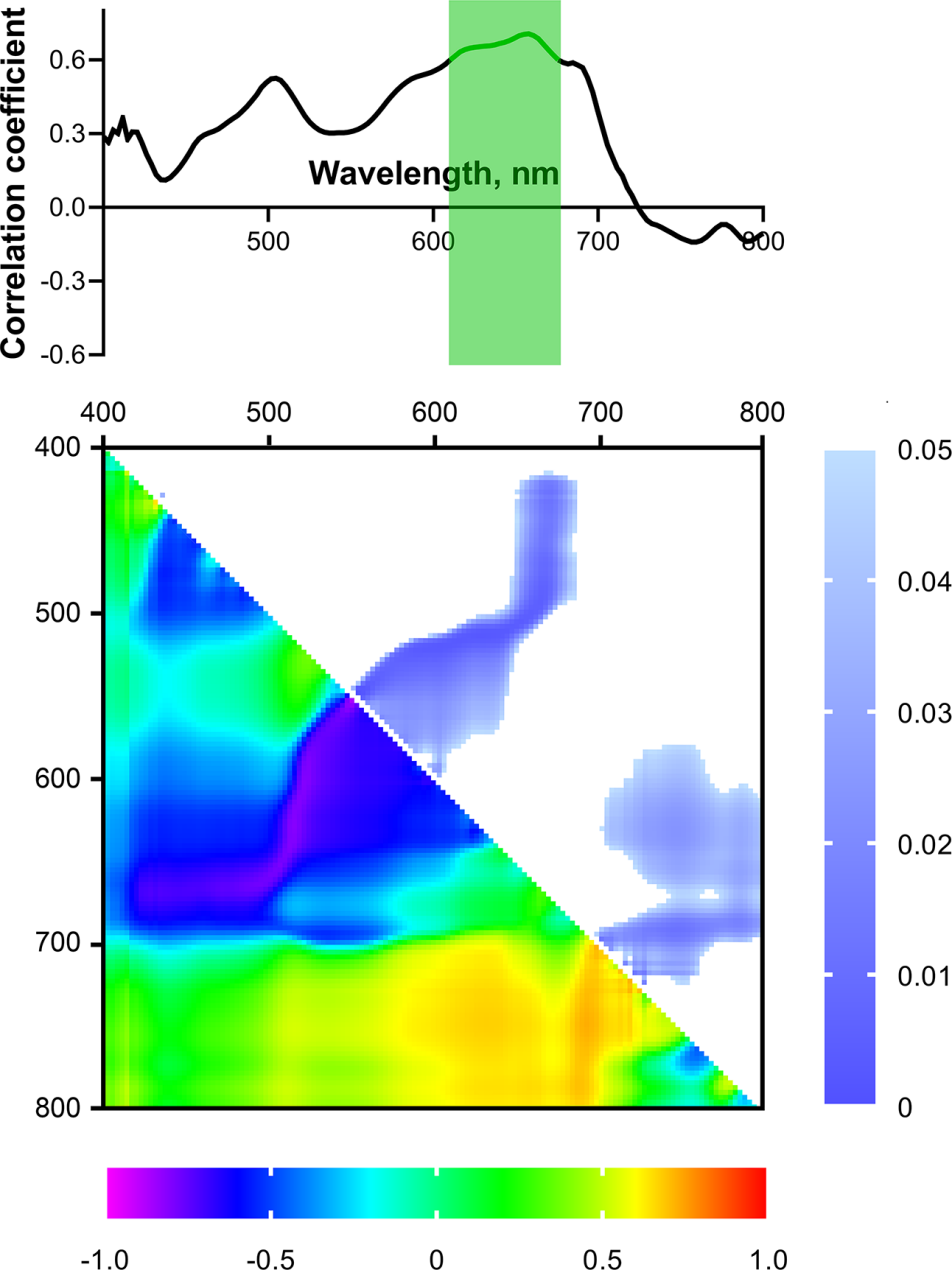
Spectrum and heat map of Pearson correlation coefficients between HS parameters of 2-week-old and heat tolerance of 4-week-old wheat plants. Area of the spectra with statistically significant correlation is highlighted in green. Heat map shows correlation coefficients (bottom left) and p-values (top right).

The hot plate temperature at which *Φ*_PSII_ decreased below the initial control value at 25°C (t_dec_) was used as a criterion for the tolerance of wheat seedlings to heat. Cultivars that exhibited later *Φ*_PSII_ suppression were considered more tolerant, and vice versa. **Figure 10** shows the spectrum of correlation coefficients between t_dec_ for 4-week-old wheat plants and reflectance in the range of 400-800 nm for 2-week-old seedlings. A significant (p< 0.05) positive correlation of these parameters was observed in the R range (610-675 nm); Pearson correlation coefficient reached 0.71.

Correlation analysis of the relationship between NDIs of 2-week-old plants and heat tolerance revealed two large areas with significant correlation on the heat map (**Figure 10**): R range [NDI(λ_1_ 580-630; λ_2_ 520-570), NDI(λ_1_ 650-690; λ_2_ 420-500)] and FR-NIR range (NDI(λ_1_ 400-700; λ_2_ 710-800)); Pearson correlation coefficients reached 0.91, 0.0.91, and –0.97, respectively (p< 0.05).

#### 3.4.2 ChlF parameters

The tolerance of 4-week-old wheat seedlings to heat, assessed by t_dec_, was positively correlated with t_1/2_(*Φ*_PSIIef_) (r = 0.66) and t(NPQ_max_) (r = 0.76), reflecting the rate of transient processes in PSA after switching on the AL (**Table 7**). No pronounced relationship with stationary parameters was observed.

**Table 7:** Correlation coefficients between ChlF parameters of 2-week-old and heat tolerance of 4-week-old wheat plants.

	F_v_/F_m_	*Φ*_PSIIef_	t_1/2_(*Φ*_PSIIef_)	NPQ_max_	t(NPQ_max_)	NPQ_s_
*r* p	*-0.33* 0.32	*-0.53* 0.10	***0.66*** **0.03**	*0.32* 0.34	***0.76*** **0.007**	*0.42* 0.20

Statistically signiﬁcant correlation coefﬁcients and the corresponding P-values (two-tailed, p< 0.05) are in bold.

## 4 Discussion

Early phenotyping-based prediction of economically important wheat traits, such as grain yield and tolerance to stressors, is a good way to develop methods for accelerating the selection of promising lines in the breeding process. In our study, the predicted traits were tolerance to heat and drought, as well as biomass accumulation, which, like yield, is largely determined by the intensity of the production process. We have previously shown that the biomass of young wheat plants correlates well with the biomass of older plants under controlled conditions (Sherstneva et al., 2021). In the field, wheat yield has been demonstrated to be related to both the biomass of mature plants (Okuyama et al., 2004; White and Wilson, 2006; Thapa et al., 2019) and the biomass of significantly younger plants, including under different conditions of water availability (Thapa et al., 2019). The presence of a relationship between yield and biomass suggests that the latter is an important target trait during phenotyping at the early stages of breeding research.

### 4.1 Relationship between spectral and fluorescent parameters in wheat seedlings

An important part of this work was the study of the relationship between reflectance parameters recorded using HS sensors and ChlF parameters recorded by PAM fluorometry. An obvious advantage of multi- and hyperspectral phenotyping methods is their high throughput and ability to perform measurements over wide spatial scales. Non-invasively obtained canopy reflectance parameters are widely used to evaluate the structural and biochemical characteristics of wheat (Al-Tamimi et al., 2022; Skendžić et al., 2023; Zhang et al., 2023). The PAM fluorometry method, which directly provides information on the activity of photosynthesis, a key physiological process that determines productivity, has both spatial and temporal limitations. In this regard, studies aimed at assessing the photosynthetic activity of plants based on reflectance parameters, as well as the search for such parameters correlating with ChlF parameters, have become widespread (Sukhova and Sukhov, 2018; Pérez-Bueno et al., 2019; Fu et al., 2022).

In our work, a number of ChlF parameters were determined and classified into two groups. The first group (stationary parameters) includes such parameters as F_v_/F_m_, *Φ*_PSIIef_, and NPQ_s_ which reflect the functioning of PSA in a state adapted to darkness or light. The second group (transient parameters) includes the parameters t_1/2_(*Φ*_PSIIef_), NPQ_max_, and t(NPQ_max_) which reflect transient processes when actinic light is switched on after dark adaptation. The relationship of the recorded ChlF parameters with the reflectance spectra of wheat leaves and NDIs, calculated from all combinations of recorded wavelengths in the range of 400-800 nm, was analyzed. Three wavelength ranges, demonstrating a strong relationship with PAM parameters, were identified (**Figure 5**): Blue (B, 420-490 nm), Red (R, 640-690 nm) and FR-NIR (710-800 nm).

F_v_/F_m_ and *Φ*_PSIIef_ (included in the group of stationary parameters) negatively correlated with the B and R bands of the VIS spectral range. It is known that leaf reflectance in VIS depends primarily on the pigment composition of the green parts of plants (Li et al., 2014; Liu et al., 2022). It can be assumed that the content of chlorophyll, which has absorption maxima in the B and R spectral bands, determines the relationship of these spectral bands with stationary ChlF parameters. The relationship between F_v_/F_m_ and *Φ*_PSIIef_ and chlorophyll content is a well-known fact (Kalaji et al., 2017).

Reflectance in the FR-NIR range was associated with ChlF parameters reflecting transient processes caused by the dark-light transition (NPQ_max_, t(NPQ_max_), t_1/2_(*Φ*_PSIIef_)). The rate of change in the *Φ*_PSII_ and NPQ parameters when actinic light is switched on is determined by the rate of achieving a balance between the production of NADPH and ATP in the light-dependent photosynthetic reactions and their consumption in the reactions of the Calvin-Benson cycle (Baker, 2008). The activity of the Calvin-Benson cycle reactions is largely determined by the availability of the substrate – CO_2_ (Baker, 2008; Flexas et al., 2008; Zhu et al., 2010; Tholen and Zhu, 2011). In this regard, it is important to note that the optical properties of the green parts of plants in the FR-NIR range, characterized by a high reflectance, are determined by the structural features of plant tissues (Ollinger, 2011). In particular, the intensity of NIR light reflection positively correlates with the volume of intercellular air spaces (Ustin and Jacquemoud, 2020), including at wavelengths in the range we studied (Slaton et al., 2001). The looser mesophyll may contribute to the greater possible rate of influx of CO_2_ consumed in cells in the Calvin-Benson cycle.

Spectral indices (NDIs), calculated for the entire studied spectral range, showed a more complex relationship with the parameters of photosynthetic activity. Two significant areas were identified on the heat maps of NDIs correlations against ChlF parameters: area for λ_1_ in R band and area for λ_1_ in FR-NIR band. The highest NDIs correlations in the R range were found for *Φ*_PSIIef_ and NPQ_s_. It is worth noting that these parameters characterize the activity of photosynthetic processes in a state adapted to light. Strong relationship of NDIs in the indicated range was also for t_1/2_(*Φ*_PSIIef_) and t(NPQ_max_); however, the size of this region was significantly smaller. The second wide area on the NDIs heat map with high correlation coefficients (FR-NIR) appeared predominantly for the parameters t_1/2_(*Φ*_PSIIef_), NPQ_max_ and t(NPQ_max_) which characterize transient processes in the photosynthetic apparatus caused by the switching on the actinic light. At the same time, stationary parameters correlated with NDIs in a much narrower range. This effect for NDIs fits into the nature of the relationship between the reflectance spectrum and the ChlF parameters, described above; in this case, R and FR-NIR can serve as measuring wavelength ranges to characterize stationary and transient processes in the photosynthetic apparatus.

The search for the relationship between NDIs in the studied wavelength range and ChlF parameters of wheat has been described in a number of works. In particular, NDVI (Normalized difference vegetation index) was significantly positively correlated with F_v_/F_m_ in wheat under optimal conditions (El-Hendawy et al., 2019). The NDVI and NDRE (Red edge NDVI) indices showed a positive relationship with F_v_/F_m_ in unstressed leaves of aspen and cherry trees (Peng et al., 2017). The work of Sukhova and Sukhov (Sukhova and Sukhov, 2020) showed that the light-induced changes in the PRIs (ΔPRIs) can be used to assess a number of parameters of PSI and PSII activity.

### 4.2 Search for predictors of biomass accumulation in wheat seedlings

During the search for predictors of biomass accumulation, a number of parameters determined by HS and PAM methods in young plants demonstrated a relationship with the biomass of older plants. Considering the spectral characteristics, we can highlight the R range (650-670 nm), which showed a significant negative correlation with DW of 4-week-old seedlings (**Figure 7**). When examining the predictive potential of spectral indices, two regions on the NDIs heat map with a significant correlation against the DW of 4-week-old seedlings were found: R and FR-NIR ranges (**Figure 7**). It is worth noting that the identified ranges of statistically significant correlations between NDIs and biomass accumulation include such widely used spectral indices as GNDVI (Green normalized difference vegetation index, (R_780_–R_550_)/(R_780_+R_550_) (Aparicio et al., 2000), and RNDVI (Red normalized difference vegetation index, (R_780_−R_670_)/(R_780_+R_670_) (Raun et al., 2001). GNDVI and RNDVI, recorded using a spectrometer with high spectral resolution, showed a high positive correlation with yield under full irrigation conditions (El-Hendawy et al., 2017). NDVI (λ_1_ 774, λ_2_ 656) recorded by a multispectral sensor was positively correlated with the yield of irrigated wheat; moreover, the yield significantly correlated with the aboveground biomass (Thapa et al., 2019). Grain yield and biomass were also positively correlated with GNDVI and RNDVI, starting from the tillering stage, under different irrigation and nitrogen levels (S. Pradhan et al., 2012). These data are consistent with the results of our work, which indicate a significant positive relationship between NDIs in FR-NIR region and biomass accumulation in wheat plants.

Correlation analysis of the relationship between potential fluorescent predictors and biomass accumulation revealed a significant correlation with stationary ChlF parameters (*Φ*_PSIIef_ and NPQ_s_), as well as with transient parameters of light-induced ChlF dynamics [t_1/2_(*Φ*_PSIIef_) and t(NPQ_max_)]. Our previous work (Sherstneva et al., 2021), carried out using other cultivars, also showed the potential of *Φ*_PSIIef_ and t_1/2_(*Φ*_PSIIef_) for predicting seedling biomass. At the same time, t_1/2_(*Φ*_PSIIef_) maintained a significant correlation with biomass accumulation with increasing prediction period.

It is worth noting that the identified HS and ChlF predictors are related to each other. The presence of such a connection suggests a generic physiological basis for the predictive potential of the fluorescent and spectral parameters used. In particular, the R range of the correlation spectrum with DW lies in the region of high correlation of the reflectance spectrum with *Φ*_PSIIef_ of plants of the same age (**Figure 5**). This relationship is consistent with the high positive correlation between these ChlF parameters and biomass accumulation 2 weeks after the ChlF recording (**Table 5**). *Φ*_PSIIef_ as a stationary parameter of photosynthetic activity in the light-adapted state reflects the efficiency of using the energy of absorbed light by photosystems and is linearly related to the rate of CO_2_ assimilation (Leipner et al., 1999; Maxwell and Johnson, 2000; Baker, 2008). These processes directly influence the synthesis of organic matter and the rate of biomass accumulation (Kruger and Volin, 2006; Ferguson et al., 2021). The parameter t_1/2_(*Φ*_PSIIef_), which characterizes the rate at which maximum quantum efficiency is achieved after actinic light is switched on, depends on the rate of activation of the Calvin-Benson cycle (Baker, 2008), which is regulated, in particular, by CO_2_ availability. The greater availability of CO_2_ determines the intensification of the production process, leading to more intensive accumulation of biomass. In the case of NDIs heat maps, the significant R region of correlation with biomass accumulation coincided to a greater extent with that for heat maps of NDIs correlations against stationary parameters of ChlF (*Φ*_PSIIef_, NPQ_s_). On the other hand, the significant region for FR-NIR range corresponded predominantly to heat maps of NDIs correlations against the transient ChlF parameters [t_1/2_(*Φ*_PSIIef_), t(NPQ_max_)] (**Figure 5**). In particular, as noted earlier, such a relationship between the ChlF parameters which characterize the rate of optimization of the dark reactions activity regulated by CO_2_ availability, and HS parameters in the FR-NIR range may be due to the structural characteristics of the leaves, in particular, the volume of intercellular air spaces (Ustin and Jacquemoud, 2020), promoting more intense gas exchange and, as a result, greater availability of CO_2_.

### 4.3 Search for predictors of tolerance of wheat seedlings to drought

The drought tolerance index (reflecting the percentage ratio between the DW of experimental and control plants) was used as the main criterion for tolerance of wheat seedlings to water deficiency. Additional tolerance criteria (residual levels of photosynthetic activity parameters in plants affected by drought, also expressed as a percentage of control levels) were also used. A statistically significant correlation between residual *Φ*_PSIIef_ and the drought tolerance index was found (**Table 3**), which is consistent with the data of earlier work, which showed the relationship between the drought tolerance index and residual *Φ*_PSIIef_ 2 weeks after stopping irrigation (Sherstneva et al., 2021). An approach to assessing drought tolerance based on the use of several criteria worked well in the search for fluorescent predictors of tolerance in wheat seedlings (Sherstneva et al., 2021) and made it possible to evaluate drought-induced suppression of physiological processes in plants. However, despite the wide range of wavelengths and high spectral resolution of the data obtained in our work, which provides high information content of the method, no strong relationship between HS and drought tolerance parameters was identified (**Figures 8**, **9**). This effect persisted for both reflectance and NDIs. Analysis of the predictive potential of ChlF parameters of 2-week-old seedlings also did not reveal their connection with tolerance to 14-day drought.

At the moment, there is a sufficient number of works devoted to the prediction of economically important traits of wheat under conditions of water deficiency (Aparicio et al., 2000; Bandyopadhyay et al., 2014; Tattaris et al., 2016; Becker and Schmidhalter, 2017; El-Hendawy et al., 2017; Condorelli et al., 2018; Thapa et al., 2019). In this case, HS parameters covering a wide range of wavelengths are used. In particular, water and other indices that take into account the reflectance in a longer wavelengths region than the range recorded in our work have shown their efficiency (Bandyopadhyay et al., 2014; Becker and Schmidhalter, 2017; El-Hendawy et al., 2017). Such indices may show a higher and more significant correlation with plant yield under water deficit conditions compared to indices in the range up to 800 nm (Becker and Schmidhalter, 2017; El-Hendawy et al., 2017). In addition, there are works showing the potential of NDVI for predicting biomass accumulation (Tattaris et al., 2016; El-Hendawy et al., 2017; Condorelli et al., 2018) and crop yield (Aparicio et al., 2000; Tattaris et al., 2016; Becker and Schmidhalter, 2017; El-Hendawy et al., 2017; Thapa et al., 2019) under conditions of water deficiency. At the same time, only a few studies used spectral predictors recorded under control conditions. In particular, the work of Condorelli et al. (Condorelli et al., 2018) showed the potential of NDVI recorded before the irrigation stop to predict wheat biomass at the terminal stage of drought. In another work, some vegetation and water indices recorded under full irrigation correlated with the yield index and a number of drought tolerance parameters of spring wheat (El-Hendawy et al., 2017).

### 4.4 Search for predictors of tolerance of wheat seedlings to heat

The next step was to assess the relationship between HS predictors and the tolerance of wheat seedlings to elevated temperatures. A fairly wide spectral band that significantly correlated with tolerance to heating two weeks after HS imaging was discovered (**Figure 10**). This range partially coincided with that for biomass accumulation under control conditions (**Figure 7**). Heat maps of correlations of NDIs with tolerance also showed partial overlap of significant regions with a heat map of correlations against biomass accumulation in the R band. The direction of the correlation for biomass accumulation and heat tolerance was opposite.

It is also important to note that ChlF parameters which have a statistically significant correlation with heat tolerance (**Table 7**), also correlated with productivity. As in the case of spectral characteristics, the correlation of these parameters with biomass and heat tolerance had the opposite trend. This result is consistent with the fact that heat tolerance was negatively correlated with biomass accumulation (r = –0.7, p = 0.02).

The discovered patterns can apparently be due to a number of causes. It is known that during heat stress, reactive oxygen species (ROS), the concentration of which increases when exposed to elevated temperatures, play a significant role in plant damage, including inactivation of photosynthesis and damage to PSA in plants (Fortunato et al., 2023). The main sites for the production of excess ROS under heat stress are the photosynthetic electron transport chain and the Calvin-Benson cycle (Allakhverdiev et al., 2008). It can be assumed that the level of ROS increases more significantly under heat stress in plants with high activity of photosynthetic processes, which ensure high productivity, compared to plants with a lower level of photosynthetic activity. A greater increase in ROS can have a greater negative effect on the PSA and, as a result, on the biomass accumulation.

## 5 Conclusion

We found that hyperspectral (HS) characteristics determined in young wheat plants can act as predictors of biomass accumulation and tolerance to heat stress. The revealed HS predictors demonstrate a statistically significant correlation with ChlF parameters which also correlate with the studied wheat traits. The presence of a relationship between HS predictors, depending on the parameters of the composition and structure of plant tissues, and ChlF predictors, depending on the activity of photosynthetic processes, increases the reliability of the prediction and allows us to reasonably assume the physiological basis of their prognostic potential. The identified potential of HS predictors determines the possibilities for accelerating the breeding process. Selection of the most promising genotypes at the early stages will significantly reduce the consumption of resources and time for further breeding trials.

Further research should focus on determination of a detailed relationship between the identified predictors and biochemical, physiological and structural parameters of plants, as well as genetic markers. Taken together, this approach will not only increase the reliability of forecasts, but will also contribute to reliable modeling of plant growth and development in various conditions based on genotyping and early phenotyping.

## Data availability statement

The original contributions presented in the study are included in the article/**Supplementary Material**. Further inquiries can be directed to the corresponding author.

## Author contributions

OS: Conceptualization, Formal Analysis, Investigation, Methodology, Writing – original draft, Writing – review & editing. FA: Visualization, Writing – review & editing. DK: Investigation, Writing – review & editing. LY: Writing – review & editing. EG: Investigation, Writing – review & editing. VV: Conceptualization, Funding acquisition, Project administration, Writing – original draft, Writing – review & editing.

## References

[B1] Allakhverdiev S. I. Kreslavski V. D. Klimov V. V. Los D. A. Carpentier R. Mohanty P. (2008). Heat stress: an overview of molecular responses in photosynthesis. Photosynth Res. 98, 541–550. doi: 10.1007/s11120-008-9331-0 18649006

[B2] Alotaibi F. Alharbi S. Alotaibi M. Al Mosallam M. Motawei M. Alrajhi A. (2021). Wheat omics: Classical breeding to new breeding technologies. Saudi J. Biol. Sci. 28, 1433–1444. doi: 10.1016/j.sjbs.2020.11.083 33613071 PMC7878716

[B3] Al-Tamimi N. Langan P. Bernád V. Walsh J. Mangina E. Negrão S. (2022). Capturing crop adaptation to abiotic stress using image-based technologies. Open Biol. 12, 210353. doi: 10.1098/rsob.210353 35728624 PMC9213114

[B4] Aparicio N. Villegas D. Casadesus J. Araus J. L. Royo C. (2000). Spectral vegetation indices as nondestructive tools for determining durum wheat yield. Agron. J. 92, 83–91. doi: 10.2134/agronj2000.92183x

[B5] Baker N. R. (2008). Chlorophyll fluorescence: A probe of photosynthesis *in vivo* . Annu. Rev. Plant Biol. 59, 89–113. doi: 10.1146/annurev.arplant.59.032607.092759 18444897

[B6] Bandyopadhyay K. K. Pradhan S. Sahoo R. N. Singh R. Gupta V. K. Joshi D. K.. (2014). Characterization of water stress and prediction of yield of wheat using spectral indices under varied water and nitrogen management practices. Agric. Water Manage. 146, 115–123. doi: 10.1016/j.agwat.2014.07.017

[B7] Becker E. Schmidhalter U. (2017). Evaluation of yield and drought using active and passive spectral sensing systems at the reproductive stage in wheat. Front. Plant Sci. 8. doi: 10.3389/fpls.2017.00379 PMC5372809 28424706

[B8] Bhandari M. Baker S. Rudd J. C. Ibrahim A. M. H. Chang A. Xue Q.. (2021). Assessing the effect of drought on winter wheat growth using unmanned aerial system (UAS)-based phenotyping. Remote Sens. 13, 1144. doi: 10.3390/rs13061144

[B9] Condorelli G. E. Maccaferri M. Newcomb M. Andrade-Sanchez P. White J. W. French A. N.. (2018). Comparative aerial and ground based high throughput phenotyping for the genetic dissection of NDVI as a proxy for drought adaptive traits in durum wheat. Front. Plant Sci. 9. doi: 10.3389/fpls.2018.00893 PMC6028805 29997645

[B10] Crain J. Mondal S. Rutkoski J. Singh R. P. Poland J. (2018). Combining high-throughput phenotyping and genomic information to increase prediction and selection accuracy in wheat breeding. Plant Genome 11, 170043. doi: 10.3835/plantgenome2017.05.0043 PMC12962554 29505641

[B11] Dos Santos T. B. Ribas A. F. De Souza S. G. H. Budzinski I. G. F. Domingues D. S. (2022). Physiological responses to drought, salinity, and heat stress in plants: A review. Stresses 2, 113–135. doi: 10.3390/stresses2010009

[B12] El-Hendawy S. Elshafei A. Al-Suhaibani N. Alotabi M. Hassan W. Dewir Y. H.. (2019). Assessment of the salt tolerance of wheat genotypes during the germination stage based on germination ability parameters and associated SSR markers. J. Plant Interact. 14, 151–163. doi: 10.1080/17429145.2019.1603406

[B13] El-Hendawy S. E. Hassan W. M. Al-Suhaibani N. A. Schmidhalter U. (2017). Spectral assessment of drought tolerance indices and grain yield in advanced spring wheat lines grown under full and limited water irrigation. Agric. Water Manage. 182, 1–12. doi: 10.1016/j.agwat.2016.12.003

[B14] Ferguson J. N. Tidy A. C. Murchie E. H. Wilson Z. A. (2021). The potential of resilient carbon dynamics for stabilizing crop reproductive development and productivity during heat stress. Plant Cell Environ. 44, 2066–2089. doi: 10.1111/pce.14015 33538010

[B15] Flexas J. Ribas-Carbó M. Diaz-Espejo A. Galmés J. Medrano H. (2008). Mesophyll conductance to CO_2_: current knowledge and future prospects. Plant Cell Environ. 31, 602–621. doi: 10.1111/j.1365-3040.2007.01757.x 17996013

[B16] Fortunato S. Lasorella C. Dipierro N. Vita F. De Pinto M. C. (2023). Redox signaling in plant heat stress response. Antioxidants 12, 605. doi: 10.3390/antiox12030605 36978852 PMC10045013

[B17] Fu P. Montes C. M. Siebers M. H. Gomez-Casanovas N. McGrath J. M. Ainsworth E. A.. (2022). Advances in field-based high-throughput photosynthetic phenotyping. J. Exp. Bot. 73, 3157–3172. doi: 10.1093/jxb/erac077 35218184 PMC9126737

[B18] Gosa S. C. Lupo Y. Moshelion M. (2019). Quantitative and comparative analysis of whole-plant performance for functional physiological traits phenotyping: New tools to support pre-breeding and plant stress physiology studies. Plant Sci. 282, 49–59. doi: 10.1016/j.plantsci.2018.05.008 31003611

[B19] Grzybowski M. Wijewardane N. K. Atefi A. Ge Y. Schnable J. C. (2021). Hyperspectral reflectance-based phenotyping for quantitative genetics in crops: Progress and challenges. Plant Commun. 2, 100209. doi: 10.1016/j.xplc.2021.100209 34327323 PMC8299078

[B20] Hassan M. A. Fei S. Li L. Jin Y. Liu P. Rasheed A.. (2022). Stacking of canopy spectral reflectance from multiple growth stages improves grain yield prediction under full and limited irrigation in wheat. Remote Sens. 14, 4318. doi: 10.3390/rs14174318

[B21] Hossain A. Skalicky M. Brestic M. Maitra S. Ashraful Alam M. Syed M. A.. (2021). Consequences and Mitigation Strategies of Abiotic Stresses in Wheat (Triticum aestivum L.) under the Changing Climate. Agronomy 11, 241. doi: 10.3390/agronomy11020241

[B22] Juliana P. Montesinos-López O. A. Crossa J. Mondal S. González Pérez L. Poland J.. (2019). Integrating genomic-enabled prediction and high-throughput phenotyping in breeding for climate-resilient bread wheat. Theor. Appl. Genet. 132, 177–194. doi: 10.1007/s00122-018-3206-3 30341493 PMC6320358

[B23] Kalaji H. M. Schansker G. Brestic M. Bussotti F. Calatayud A. Ferroni L.. (2017). Frequently asked questions about chlorophyll fluorescence, the sequel. Photosynth Res. 132, 13–66. doi: 10.1007/s11120-016-0318-y 27815801 PMC5357263

[B24] Katsoulas N. Elvanidi A. Ferentinos K. P. Kacira M. Bartzanas T. Kittas C. (2016). Crop reflectance monitoring as a tool for water stress detection in greenhouses: A review. Biosyst. Eng. 151, 374–398. doi: 10.1016/j.biosystemseng.2016.10.003

[B25] Kim M. Lee C. Hong S. Kim S. L. Baek J.-H. Kim K.-H. (2021). High-throughput phenotyping methods for breeding drought-tolerant crops. IJMS 22, 8266. doi: 10.3390/ijms22158266 34361030 PMC8347144

[B26] Krause M. R. González-Pérez L. Crossa J. Pérez-Rodríguez P. Montesinos-López O. Singh R. P.. (2019). Hyperspectral reflectance-derived relationship matrices for genomic prediction of grain yield in wheat. G3 Genes|Genomes|Genetics 9, 1231–1247. doi: 10.1534/g3.118.200856 30796086 PMC6469421

[B27] Kruger E. L. Volin J. C. (2006). Reexamining the empirical relation between plant growth and leaf photosynthesis. Funct. Plant Biol. 33, 421. doi: 10.1071/FP05310 32689249

[B28] Leipner J. Fracheboud Y. Stamp P. (1999). Effect of growing season on the photosynthetic apparatus and leaf antioxidative defenses in two maize genotypes of different chilling tolerance. Environ. Exp. Bot. 42, 129–139. doi: 10.1016/S0098-8472(99)00026-X

[B29] Lesk C. Anderson W. Rigden A. Coast O. Jägermeyr J. McDermid S.. (2022). Compound heat and moisture extreme impacts on global crop yields under climate change. Nat. Rev. Earth Environ. 3, 872–889. doi: 10.1038/s43017-022-00368-8

[B30] Li L. Zhang Q. Huang D. (2014). A review of imaging techniques for plant phenotyping. Sensors 14, 20078–20111. doi: 10.3390/s141120078 25347588 PMC4279472

[B31] Lippmann R. Babben S. Menger A. Delker C. Quint M. (2019). Development of wild and cultivated plants under global warming conditions. Curr. Biol. 29, R1326–R1338. doi: 10.1016/j.cub.2019.10.016 31846685

[B32] Liu S. Hu Z. Han J. Li Y. Zhou T. (2022). Predicting grain yield and protein content of winter wheat at different growth stages by hyperspectral data integrated with growth monitor index. Comput. Electron. Agric. 200, 107235. doi: 10.1016/j.compag.2022.107235

[B33] Lozada D. N. Carter A. H. (2020). Genomic selection in winter wheat breeding using a recommender approach. Genes 11, 779. doi: 10.3390/genes11070779 32664601 PMC7397162

[B34] Mao H. Jiang C. Tang C. Nie X. Du L. Liu Y.. (2023). Wheat adaptation to environmental stresses under climate change: Molecular basis and genetic improvement. Mol. Plant 16, 1564–1589. doi: 10.1016/j.molp.2023.09.001 37671604

[B35] Maxwell K. Johnson G. N. (2000). Chlorophyll fluorescence—a practical guide. J. Exp. Bot. 51, 659–668. doi: 10.1093/jexbot/51.345.659 10938857

[B36] Ni Z. Lu Q. Huo H. Zhang H. (2019). Estimation of chlorophyll fluorescence at different scales: A review. Sensors 19, 3000. doi: 10.3390/s19133000 31288380 PMC6651496

[B37] Okuyama L. A. Federizzi L. C. Barbosa Neto J. F. (2004). Correlation and path analysis of yield and its components and plant traits in wheat. Cienc. Rural 34, 1701–1708. doi: 10.1590/S0103-84782004000600006

[B38] Ollinger S. V. (2011). Sources of variability in canopy reflectance and the convergent properties of plants. New Phytol. 189, 375–394. doi: 10.1111/j.1469-8137.2010.03536.x 21083563

[B39] Peng Y. Zeng A. Zhu T. Fang S. Gong Y. Tao Y.. (2017). Using remotely sensed spectral reflectance to indicate leaf photosynthetic efficiency derived from active fluorescence measurements. J. Appl. Remote Sens 11, 26034. doi: 10.1117/1.JRS.11.026034

[B40] Pérez-Bueno M. L. Pineda M. Barón M. (2019). Phenotyping plant responses to biotic stress by chlorophyll fluorescence imaging. Front. Plant Sci. 10. doi: 10.3389/fpls.2019.01135 PMC6759674 31620158

[B41] Perez-Sanz F. Navarro P. J. Egea-Cortines M. (2017). Plant phenomics: an overview of image acquisition technologies and image data analysis algorithms. GigaScience 6, 1–18. doi: 10.1093/gigascience/gix092 PMC5737281 29048559

[B42] Pradhan S. Bandyopadhyay K. K. Joshi D. K. (2012). Canopy reflectance spectra of wheat as related to crop yield, grain protein under different management practices. J. Agrometeorol. 14, 21–25. doi: 10.54386/jam.v14i1.1373

[B43] Raun W. R. Solie J. B. Johnson G. V. Stone M. L. Lukina E. V. Thomason W. E.. (2001). In-season prediction of potential grain yield in winter wheat using canopy reflectance. Agron. J. 93, 131–138. doi: 10.2134/agronj2001.931131x

[B44] Rutkoski J. Poland J. Mondal S. Autrique E. Pérez L. G. Crossa J.. (2016). Canopy temperature and vegetation indices from high-throughput phenotyping improve accuracy of pedigree and genomic selection for grain yield in wheat. G3 Genes|Genomes|Genetics 6, 2799–2808. doi: 10.1534/g3.116.032888 27402362 PMC5015937

[B45] Sánchez-Bermúdez M. Del Pozo J. C. Pernas M. (2022). Effects of combined abiotic stresses related to climate change on root growth in crops. Front. Plant Sci. 13. doi: 10.3389/fpls.2022.918537 PMC9284278 35845642

[B46] Sarić R. Nguyen V. D. Burge T. Berkowitz O. Trtílek M. Whelan J.. (2022). Applications of hyperspectral imaging in plant phenotyping. Trends Plant Sci. 27, 301–315. doi: 10.1016/j.tplants.2021.12.003 34998690

[B47] Shahzad A. Ullah S. Dar A. A. Sardar M. F. Mehmood T. Tufail M. A.. (2021). Nexus on climate change: agriculture and possible solution to cope future climate change stresses. Environ. Sci. pollut. Res. 28, 14211–14232. doi: 10.1007/s11356-021-12649-8 33515149

[B48] Sharma D. K. Andersen S. B. Ottosen C. Rosenqvist E. (2015). Wheat cultivars selected for high F _v_/F _m_ under heat stress maintain high photosynthesis, total chlorophyll, stomatal conductance, transpiration and dry matter. Physiologia Plantarum 153, 284–298. doi: 10.1111/ppl.12245 24962705

[B49] Sherstneva O. Khlopkov A. Gromova E. Yudina L. Vetrova Y. Pecherina A.. (2021). Analysis of chlorophyll fluorescence parameters as predictors of biomass accumulation and tolerance to heat and drought stress of wheat (. Funct. Plant Biol. 49, 155–169. doi: 10.1071/FP21209 34813421

[B50] Shiferaw B. Smale M. Braun H.-J. Duveiller E. Reynolds M. Muricho G. (2013). Crops that feed the world 10. Past successes and future challenges to the role played by wheat in global food security. Food Sec. 5, 291–317. doi: 10.1007/s12571-013-0263-y

[B51] Skendžić S. Zovko M. Lešić V. Pajač Živković I. Lemić D. (2023). Detection and evaluation of environmental stress in winter wheat using remote and proximal sensing methods and vegetation indices—A review. Diversity 15, 481. doi: 10.3390/d15040481

[B52] Slaton M. R. Raymond Hunt E. Smith W. K. (2001). Estimating near-infrared leaf reflectance from leaf structural characteristics. Am. J. Bot. 88, 278–284. doi: 10.2307/2657019 11222250

[B53] Sukhova E. Sukhov V. (2018). Connection of the photochemical reflectance index (PRI) with the photosystem II quantum yield and nonphotochemical quenching can be dependent on variations of photosynthetic parameters among investigated plants: A meta-analysis. Remote Sens. 10, 771. doi: 10.3390/rs10050771

[B54] Sukhova E. Sukhov V. (2020). Relation of photochemical reflectance indices based on different wavelengths to the parameters of light reactions in photosystems I and II in pea plants. Remote Sens. 12, 1312. doi: 10.3390/rs12081312

[B55] Sun J. Rutkoski J. E. Poland J. A. Crossa J. Jannink J. Sorrells M. E. (2017). Multitrait, random regression, or simple repeatability model in high-throughput phenotyping data improve genomic prediction for wheat grain yield. Plant Genome 10. doi: 10.3835/plantgenome2016.11.0111 28724067

[B56] Tattaris M. Reynolds M. P. Chapman S. C. (2016). A direct comparison of remote sensing approaches for high-throughput phenotyping in plant breeding. Front. Plant Sci. 7. doi: 10.3389/fpls.2016.01131 PMC4971441 27536304

[B57] Terletskaya N. V. Stupko V. Y. Altayeva N. A. Kudrina N. O. Blavachinskaya I. V. Kurmanbayeva M. S.. (2021). Photosynthetic activity of Triticum dicoccum × Triticum aestivum alloplasmic lines during vegetation in connection with productivity traits under varying moister conditions. Photosynt 59, 74–83. doi: 10.32615/ps.2021.003

[B58] Thapa S. Rudd J. C. Xue Q. Bhandari M. Reddy S. K. Jessup K. E.. (2019). Use of NDVI for characterizing winter wheat response to water stress in a semi-arid environment. J. Crop Improvement 33, 633–648. doi: 10.1080/15427528.2019.1648348

[B59] Tholen D. Zhu X.-G. (2011). The mechanistic basis of internal conductance: A theoretical analysis of mesophyll cell photosynthesis and CO _2_ diffusion. Plant Physiol. 156, 90–105. doi: 10.1104/pp.111.172346 21441385 PMC3091052

[B60] Trono D. Pecchioni N. (2022). Candidate genes associated with abiotic stress response in plants as tools to engineer tolerance to drought, salinity and extreme temperatures in wheat: an overview. Plants 11, 3358. doi: 10.3390/plants11233358 36501397 PMC9737347

[B61] Ustin S. L. Jacquemoud S. (2020). “How the optical properties of leaves modify the absorption and scattering of energy and enhance leaf functionality,” in Remote Sensing of Plant Biodiversity. Eds. Cavender-Bares J. Gamon J. A. Townsend P. A. (Cham: Springer International Publishing), 349–384. doi: 10.1007/978-3-030-33157-3_14

[B62] Venkatesh K. Senthilkumar K. M. Mamrutha H. M. Singh G. Singh G. P. (2022). “High-temperature stress in wheat under climate change scenario, effects and mitigation strategies,” in Climate Change and Crop Stress (Cambridge, MA: Elsevier), 209–229. doi: 10.1016/B978-0-12-816091-6.00014-6

[B63] White E. M. Wilson F. E. A. (2006). Responses of grain yield, biomass and harvest index and their rates of genetic progress to nitrogen availability in ten winter wheat varieties. Irish J. Agric. Food Res. 45, 85–101.

[B64] Zahra N. Hafeez M. B. Wahid A. Al Masruri M. H. Ullah A. Siddique K. H. M.. (2023). Impact of climate change on wheat grain composition and quality. J. Sci. Food Agric. 103, 2745–2751. doi: 10.1002/jsfa.12289 36273267

[B65] Zahra N. Wahid A. Hafeez M. B. Ullah A. Siddique K. H. M. Farooq M. (2021). Grain development in wheat under combined heat and drought stress: Plant responses and management. Environ. Exp. Bot. 188, 104517. doi: 10.1016/j.envexpbot.2021.104517

[B66] Zhang H. Wang L. Jin X. Bian L. Ge Y. (2023). High-throughput phenotyping of plant leaf morphological, physiological, and biochemical traits on multiple scales using optical sensing. Crop J. 11, 1303–1318. doi: 10.1016/j.cj.2023.04.014

[B67] Zhu X.-G. Long S. P. Ort D. R. (2010). Improving photosynthetic efficiency for greater yield. Annu. Rev. Plant Biol. 61, 235–261. doi: 10.1146/annurev-arplant-042809-112206 20192734

