# Supplementary material for: Prediction of biomass accumulation and tolerance of wheat seedlings to drought and elevated temperatures using hyperspectral imaging
Source: Front Plant Sci. 2024 Feb 2;15:1344826. doi: 10.3389/fpls.2024.1344826 (PMC10869465; doi:10.3389/fpls.2024.1344826)
Supplement: Supplementary file 1 [file DataSheet_1.docx]

Supplementary Material

Prediction of Biomass Accumulation and Tolerance of Wheat Seedlings to Drought and Elevated Temperatures using Hyperspectral Imaging

Oksana Sherstneva*, Firuz Abdullaev, Dmitry Kior, Lyubov Yudina, Ekaterina Gromova, Vladimir Vodeneev

*** Correspondence:** Oksana Sherstneva: sherstneva-oksana@yandex.ru

# Supplementary Figures and Tables

## Supplementary Figures


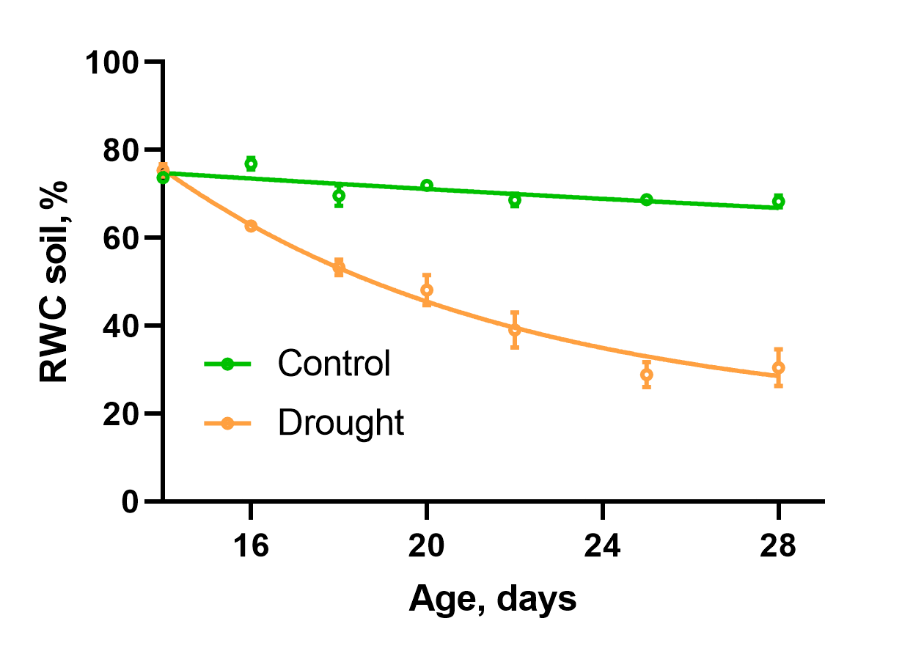


**Supplementary Figure S1.** The dynamics of relative water content (RWC) in the soil for the control and drought-stressed groups.


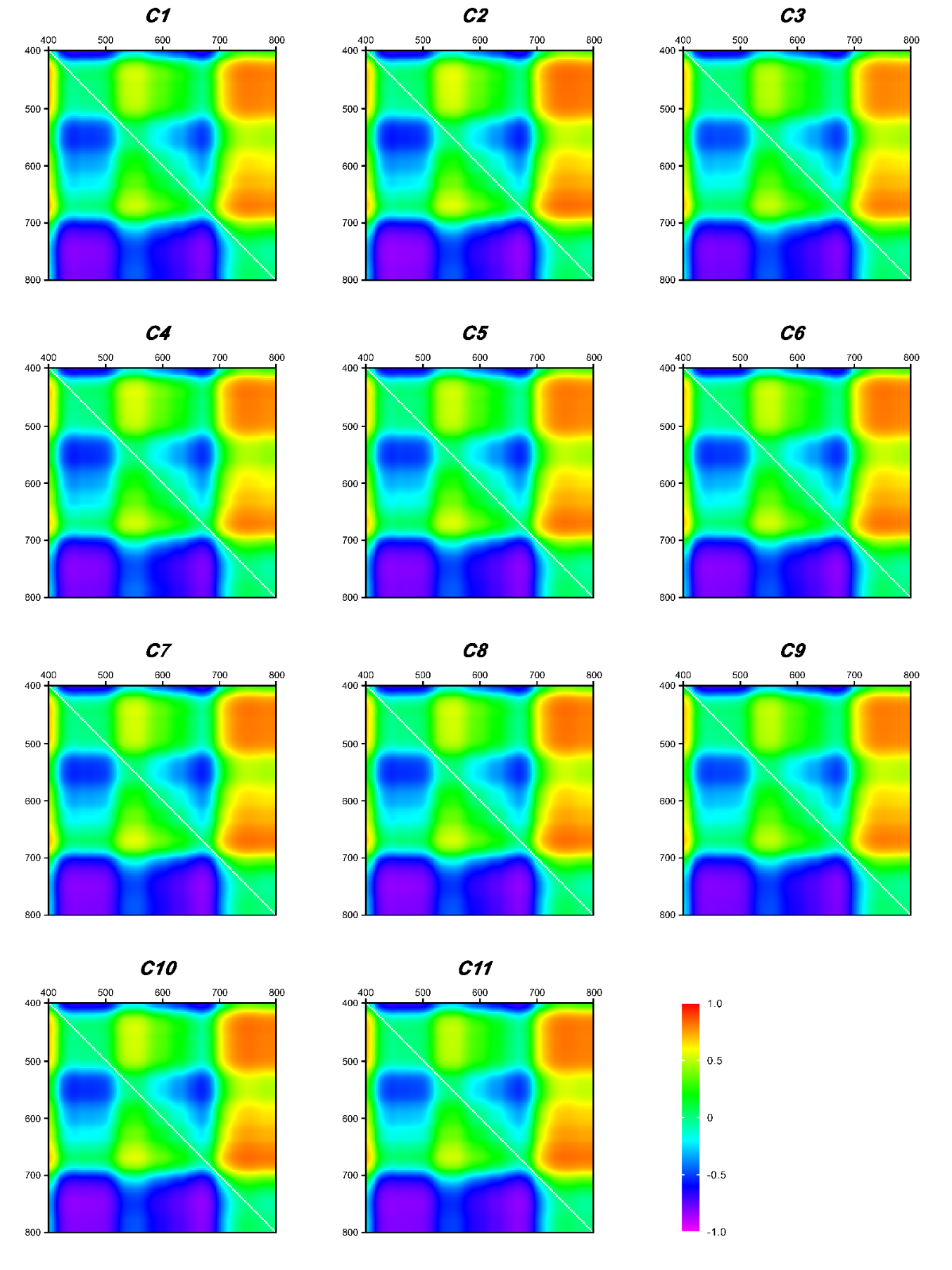


**Supplementary Figure S2.** Heat maps of NDIs of 2-week-old wheat seedlings of 11 cultivars (C1-C11).


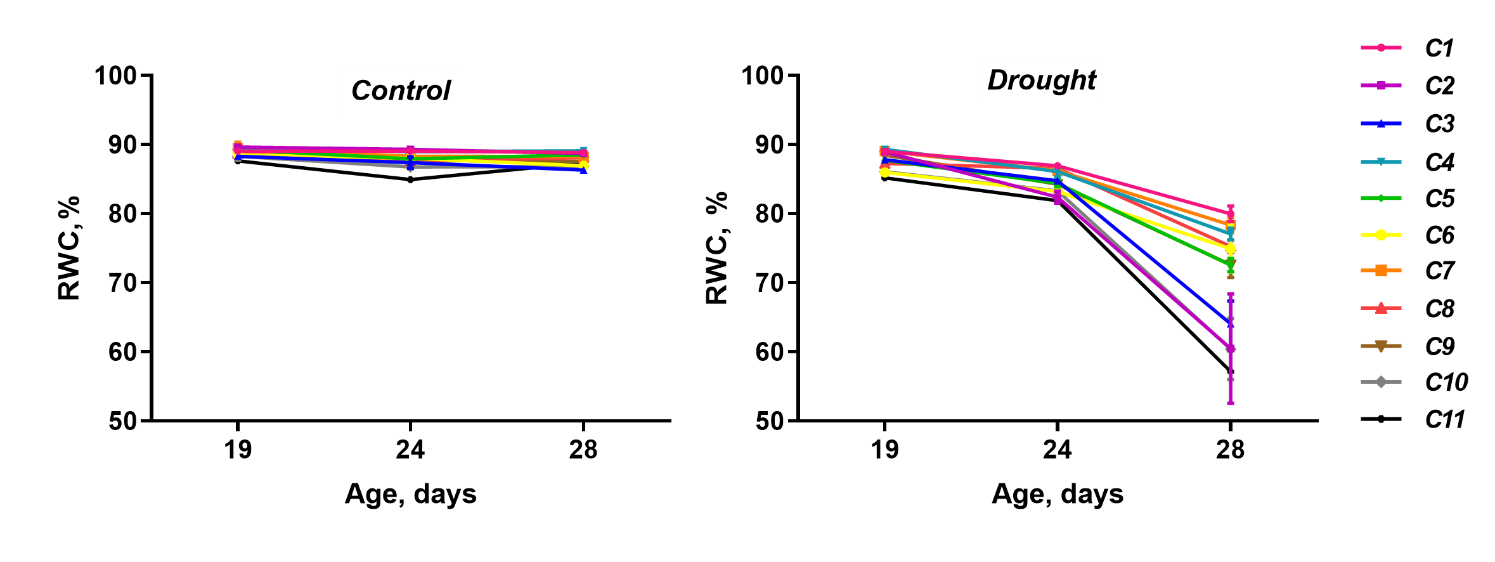


**Supplementary Figure S3.** The dynamics of relative water content (RWC) in the control and drought-stressed plants of 11 cultivars (C1-C11).

|  | **FW shoot, mg** | **DW shoot, mg** | **FW root, mg** | **DW root, mg** | **FW plant, mg** | **DW plant, mg** | **Shoot length, cm** | **Root length, cm** | **Plant length, cm** |
| --- | --- | --- | --- | --- | --- | --- | --- | --- | --- |
| C1 | 399.6±45.8 | 42.8±4.3 | 22.2±4.2 | 3.8±0.2 | 421.8±47.7 | 46.6±4.2 | 38.5±1 | 11.1±0.5 | 49.6±1.3 |
| C2 | 543.3±10.1 | 58.8±1.4 | 40.2±4.8 | 7±0.7 | 583.5±7.6 | 65.7±1.3 | 40.8±1.1 | 13.7±0.6 | 54.5±1.1 |
| C3 | 343.6±1.9 | 45.8±0.5 | 12.7±1.7 | 3.8±0.8 | 356.4±1.7 | 49.7±1.2 | 35.2±0.8 | 14.2±0.7 | 49.4±1.2 |
| C4 | 341.5±38.3 | 35.9±4.1 | 18.5±2.6 | 3.4±0.4 | 360±37.4 | 39.2±4.2 | 34.3±1.6 | 13.1±0.9 | 47.4±1.7 |
| C5 | 479.8±7.6 | 51.8±2.4 | 25.4±5.2 | 6.1±1.4 | 505.1±12.7 | 57.9±2.5 | 36.1±1.1 | 15.4±1.1 | 51.5±1.7 |
| C6 | 409.9±17.4 | 51.7±4.2 | 30.7±4.4 | 6.1±1.4 | 440.6±14.9 | 57.7±3.3 | 39.6±1.3 | 11.2±0.6 | 50.8±1.5 |
| C7 | 571.6±57.5 | 56.4±4 | 26.2±4.6 | 7±1.8 | 597.8±59.4 | 63.3±2.3 | 38.3±1 | 15.8±1 | 54.2±1.7 |
| C8 | 434.6±20.3 | 50.9±1.7 | 42.4±6.7 | 5.5±0.5 | 477±26.3 | 56.4±1.5 | 33.3±1.1 | 15.4±0.7 | 48.7±1.6 |
| C9 | 383.2±26.3 | 44.8±1.8 | 29.9±9.4 | 5.8±1 | 413±34.3 | 50.6±2.5 | 32±0.9 | 12.9±0.6 | 45±1.2 |
| C10 | 416.4±22.6 | 56.7±5.5 | 32.1±9.5 | 6.9±1.4 | 448.5±28.3 | 63.6±5.1 | 35.7±0.8 | 15.8±0.4 | 51.6±1.1 |
| C11 | 460.1±14.8 | 56.9±2.5 | 32.4±5.1 | 5.4±0.8 | 492.5±11.4 | 62.4±3.2 | 33±0.9 | 12.9±0.7 | 45.9±1.3 |

## Supplementary Tables

**Supplementary Table S1.** Morphological traits of 4-week-old wheat plants

FW - fresh weight, DW - dry weight. Data are represented as mean ± SEM. Two pots of 9 plants for each cultivar were assessed. Weight was assessed integrally for 4 or 5 plants in the pot and calculated for an individual plant (*n = 4*); length was measured for each plant (*n = 18*).

**Supplementary Table S2.** Residual values of morphological traits, expressed in % of control, after 14-d drought stress of 4-week-old wheat plants

|  | **Residual FW shoot, %** | **Residual DW shoot, %** | **Residual FW root, %** | **Residual DW root, %** | **Residual FW plant, %** | **Residual DW plant, %** |
| --- | --- | --- | --- | --- | --- | --- |
| C1 | 37.2±3.3 | 74.8±0.3 | 128.7±21.0 | 162.1±6.4 | 42.0±3.9 | 81.9±0.4 |
| C2 | 19.9±3.7 | 67.9±2.1 | 44.8±10.9 | 68.6±16.3 | 21.6±3.7 | 68.0±3.3 |
| C3 | 26.4±3.4 | 70.2±4.2 | 123.9±18.5 | 130.4±8.5 | 29.8±3.4 | 74.8±4.4 |
| C4 | 48.7±4.3 | 100.6±4.6 | 106.9±19.3 | 185.2±34.8 | 51.7±4.5 | 107.9±6.3 |
| C5 | 37.3±1.4 | 90.1±5.0 | 81.9±8.1 | 134.9±29.4 | 39.5±1.6 | 94.9±6.9 |
| C6 | 48.5±7.4 | 88.8±1.7 | 67.0±6.2 | 100.2±16.3 | 49.8±7.0 | 90.0±2.1 |
| C7 | 32.5±1.6 | 70.1±3.2 | 110.7±15.1 | 106.0±16.0 | 35.9±1.6 | 74.0±3.7 |
| C8 | 36.3±2.1 | 75.1±3.5 | 62.3±10.0 | 133.0±24.7 | 38.6±2.3 | 80.7±5.2 |
| C9 | 34.9±3.5 | 81.8±6.7 | 67.8±6.6 | 89.3±19.2 | 37.3±3.2 | 82.6±6.2 |
| C10 | 19.3±3.5 | 64.6±5.7 | 99.2±14.1 | 95.3±10.3 | 25.0±2.8 | 67.9±5.0 |
| C11 | 25.3±2.6 | 87.4±4.5 | 79.5±12.7 | 142.8±14.2 | 28.8±1.9 | 92.2±3.1 |

FW - fresh weight, DW - dry weight. Data are represented as mean ± SEM (n = 4).
